# Pooled cross-sectional sample data of the 2015, 2016, 2017 National Health Interview Surveys for studying the determinants of health care market and labor market outcomes in post Affordable Care Act USA

**DOI:** 10.1016/j.dib.2018.10.130

**Published:** 2018-10-30

**Authors:** Ibrahim Niankara

**Affiliations:** College of Business, Al Ain University of Science and Technology, Abu Dhabi, UAE

## Abstract

This data article contains health care market and labor market outcome measures along with their determinants for individuals in the civilian non-institutionalized population of the United States. The underlying source data is the anonymized person׳s level publically released versions of the 2015, 2016 and 2017 National Health Interview Surveys (NHIS). The included Health care market outcomes are “Access to health care”, “Usage of Health care”, and “Level of Expenditure on health care”; while the labor market outcomes are “Employment Status”,“ Full-time participation Status”, and “Level of hours spent working”. Together, these outcome measures describe the intensive and extensive margins of individual׳s Health care demand and labor supply in the United States. Although the original paper [4] focused on investigating the relative influence of inter-generational co-residence on these outcomes for the year 2015 and 2016, this full data set includes additional information for the year 2017 allowing one to investigate further interesting aspects of the US health care market and labor market for the period post Affordable Care Act. The determinant factors covered in the data include not only socio- economic and demographic characteristics but also key policy variables such as “Extended coverage status” and “Flexible account coverage status”.

**Specifications table**

**Table:** 

Subject area	*Economics*
More specific subject area	*Labor and Health Economics*
Type of data	*Table*
How data was acquired	*From the anonymized person׳s level publically released 2015, 2016, and 2017 data files of the National Health Interview Survey (NHIS)*
Data format	*R format data*
Experimental factors	*Health care market Outcomes and Determinants, Labor Market Outcomes and Determinants*
Experimental features	*Repeated cross-sectional random samples with individual level final weights that include post-stratification adjustments (age, race/ethnicity, sex) using US Census Bureau population control totals.*
Data source location	*The United States of America (USA), All four census regions of the country*
Data accessibility	*The Data is with this article*
Related research article	“The relative influence of inter-generational co-residence on healthcare market and labor market outcomes in post-Affordable Care Act USA”
	*Forthcoming in the Journal “Global Business and Economics Review”, see:*http://www.inderscience.com/info/ingeneral/forthcoming.php?jcode=GBER

**Value of the data**

• This data allows researchers to quickly examine individual health care market and labor market behaviors in the period post Affordable Care Act (ACA) enforcement in the US, using a nationally representative sample, without having to go through the tedious process of data acquisition and treatment from the raw public files.
• The data provides the opportunity to not only look at each market separately, but also to investigate their inter-dependence at the individual level depending on the formulated study goal.
• This data could also inspire other works, which might consider expending the data coverage to the period pre-ACA for a much richer comparative analysis of ACA effects on health care market and labor market outcomes using evidence from the National Health Interview Surveys.

## 1 Data

The data provided and described in this paper is extracted from the raw National Health Interview Survey (NHIS), which is a cross-sectional household multistage probability sample survey, conducted annually by interviewers of the US Census Bureau for the Centers for Disease Control and Prevention׳s National Center for Health Statistics (NCHS). Further details on its sampling design are found in the NCHS report [5]. The target population is the civilian non-institutionalized population residing in the USA at the time of the interview.

Each annual NHIS questionnaire (also called the core) consists of four main components: household composition section, family core, sample adult core and sample child core. The current data is from the sample adult core, which obtains additional information on health conditions, activity limitations, health behaviors and access to and use of healthcare services from one randomly selected adult (the “sample adult”) in the family. The publicly released data files (also called “public use data file”) for the 2015, 2016 and 2017 NHIS are described in Table 1.

**Table 1:** Publicly released data files characteristics for the “family” and “sample core” components.

**Year**	**2015**	**2016**	**2017**
Number of households interviewed (a)	41493	40220	32617
Number of persons interviewed (b)	103789	97169	78543
Number of families interviewed (c)	42288	40875	33157
Total households response rate (d)	70.1%	67.9%	66.5%
$\mathrm{Conditional family response rate}=\frac{c}{\mathrm{Total\backslash Eligible\backslash Families}}$ × 100 (e)	98.9%	98.9%	98.9%
Final family response rate = e × c (f)	69.3%	67.1%	65.7%
$\mathrm{Conditional adult core response rate}=\frac{b}{\mathrm{Total\backslash Eligible\backslash Sample\backslash Adults}}$ ×100 (g)	79.7%	80.9%	80.7%
Final adult core response rate = f × g (h)	55.2%	54.3%	53.0%

After variables selection, data treatment, and dropping observations (respondents) with missing information, the resulting pooled cross-sectional panel data is described next in Table 2.

**Table 2:** Characteristics of the final treated and shared data files.

**Year**	**2015**	**2016**	**2017**	**Total**
**Final number of sample core adults** (i)	16,028	16,369	13,635	46,032
Initial number of Sample core adults = c (j)	42,288	40,875	33,157	116,320
Retention Rate = $\frac{i}{j}$ ×100 (k)	37.90%	40.05%	41.12%	39.57%

Table 3 below describes the key quantitative variables in the dataset, while (Table 4a–e) describe the qualitative variables.

**Table 3:** Quantitative variables description.

**Variable**	**Description**	**Units**	**2015 (N1 = 16,028)**		**2016 (N2 = 16,369)**		**2017 (N3 = 13,635)**		**Total (N = 46,032)**	
			Mean	s.d.	Mean	s.d.	Mean	s.d.	Mean	s.d.
WRKHRS2	Hours Worked	(in a week)	42.62	12.29	41.20	12.14	41.61	12.38	41.47	12.27
AGE_P	AGE	(in years)	44.11	12.83	44.72	13.05	44.74	13.13	44.51	13.00
EDUC1	Education	(in levels)	16.11	2.68	16.19	2.58	16.35	2.47	16.21	2.59
HICOSTR1	Annual Premium Cost	(in $100)	38.76	34.70	40.24	35.74	41.45	37.33	40.08	35.88
WTFA	Respondent Final Annual Weight in the Data	(in 100)	32.05	22.59	32.78	21.38	42.25	18.98	35.03	21.52

**Note**: s.d. represents the standard deviation of the quantitative variable.

**Table 4:** Qualitative variables description.

**Variable**	**Description**	**Units**	**2015 (N1 = 16,028)**		**2016 (N2 = 16,369)**		**2017 (N3 = 13,635)**		**Total (N = 46,032)**	
			Freq.	Rel. Freq.	Freq.	Rel. Freq.	Freq.	Rel. Freq.	Freq.	Rel. Freq.
Coresid	Inter-generational co-residence Status	0: no	14,638	91.3	15,002	91.6	12,442	91.3	42,082	91.4
		1: yes	1390	8.7	1367	8.4	1193	8.7	3950	8.6
Access	Access to care despite costs	0: no	484	3	540	3.3	401	2.9	1425	3.1
		1: yes	15,544	97	15,829	96.7	13,234	97.1	44,607	96.9
Usage	USAGE 10 times or more in 12 months	0: no	14,681	91.6	14,912	91.1	12,379	90.8	41,972	91.2
		1: yes	1347	8.4	1457	8.9	1256	9.2	4060	8.8
FamMedExp	Medical Expenditure in past 12 months	1: [$0 – $499]	5431	33.9	5301	32.4	4298	31.5	15,030	32.7
		2: [$500–1999]	5712	35.6	5658	34.6	4645	34.1	16,015	34.8
		3: $2000 and more	4885	30.5	5410	33.1	4692	34.4	14,987	32.6
WorkStat	Respondent work status	0: Not working	15,339	4.3	15,677	4.2	13,049	95.7	44,065	95.7
		1: yes working for pay	689	95.7	692	95.8	586	4.3	1967	4.3
Parttime	Worked Hours per week (Binary)	0: Part-time (< 40 h)	3369	21	3639	22.2	2893	21.2	9901	21.5
		1: Full-time (≥ 40 h)	12,659	79	12,730	77.8	10,742	78.8	36,131	78.5
Parttime1	Worked Hours per week (Multinomial)	1: Less than 40 h	3369	21.0	3639	22.2	2893	21.2	9901	21.5
		2: [40–80 h.]	1,2476	77.8	12,568	76.8	10,569	77.5	35,613	77.4
		3: more than 80 h	183	1.1	162	1.0	173	1.3	518	1.1
Earning2	Annual earnings	1: $0–34,999	5581	34.8	5372	32.8	4151	30.4	15,104	32.8
		2: $35,000–64,999	5663	35.3	5821	35.6	4813	35.3	16,297	35.4
		3: 65,000 and +	4783	29.8	5176	31.6	4671	34.3	14,631	31.8
SRVY_YR	Survey collection year	1: 2015	16,028	100	–	–	–	–	16,028	34.8
		2: 2016	–	–	16,369	100	–	–	16,369	35.6
		3: 2017	–	–	–	–	13,635	100	13,635	29.6
SEX	Respondent Gender	1: Male	8189	51.1	8266	50.5	6929	50.8	23,384	50.8
		2: Female	7839	48.9	8103	49.5	6706	49.2	22,648	49.2
RACRECI3	Respondent Race	1: White	12,955	80.8	13,870	84.7	11,438	83.9	38,263	83.1
		2: Black	1744	10.9	1358	8.3	1192	8.7	4294	9.3
		3: Asian	1147	7.2	943	5.8	847	6.2	2937	6.4
		4: All other races	182	1.1	198	1.2	158	1.2	538	1.2
MaritStat	Respondent Marital Status	1: Currently married	10,460	65.3	10,840	66.2	8913	65.4	30,213	65.6
		2: Previously Married	1901	11.9	1949	11.9	1641	12.0	5491	11.9
		3: Never Married	3667	22.9	3580	21.9	3081	22.6	10,328	22.4
REGION2	Region of residency	1: South	5395	33.7	5422	33.1	4819	35.3	15,636	34.0
		2: Northwest	2650	16.5	2857	17.5	2251	16.5	7758	16.9
		3: Midwest	3680	23.0	3904	23.8	3499	25.7	11,083	24.1
		4: West	4303	26.8	4186	25.6	3066	22.5	11,555	25.1
PLBORN	Place of Birth	1: Born in the USA	13,287	82.9	14,144	86.4	11,752	86.2	39,183	85.1
		2: Born Outside USA	2741	17.1	2225	13.6	1883	13.8	6849	14.9
CITIZENP	Citizenship status	1: US Citizen	15,061	94	15,578	95.2	13,046	95.7	43,685	94.9
		2: Not US Citizen	967	6	791	4.8	589	4.3	2347	5.1
MEDBILL	Have problems paying medical bills	1: yes	2014	12.6	2000	12.2	1624	11.9	5638	12.2
		2: No	14,014	87.4	14,369	87.8	12,011	88.1	40,394	87.8
MEDBPAY	Medical Bills being paid off overtime	1: Yes	4336	27.1	4391	26.8	3666	26.9	12,393	26.9
		2: No	11,692	72.9	11,978	73.2	9969	73.1	33,639	73.1
FSA	Have a flexible spending account	1: yes have	3749	23.4	3977	24.3	3418	25.1	11144	24.2
		2: no do not have	12,279	76.6	12,392	75.7	10,217	74.9	34,888	75.8
PHSTAT2	Physical Health Status	1: Excellent	5404	33.7	5366	32.8	4501	33.0	15,271	33.2
		2: Very Good	6027	37.6	6437	39.3	5386	39.5	17,850	38.8
		3: Good	3835	23.9	3798	23.2	3092	22.7	10,725	23.3
		4: Fair or Poor	762	4.8	768	4.7	656	4.8	2186	4.7
PLIMANY2	Limited in anyway Physical, Mental, or Emotional	1: Yes	576	3.6	707	4.3	596	4.4	1879	4.1
		2: no	15,452	96.4	15,662	95.7	13,039	95.6	44,153	95.9
SINGLE2	Single Plan (Optional Insurance coverage)	1: Yes	8740	54.5	9580	58.5	8132	59.6	26452	57.5
		2: no	7288	45.5	6789	41.5	5503	40.4	19580	42.5
PHOSPYR2	Had a hospital visit over the past 12 Months	1: yes	872	5.4	893	5.5	752	5.5	2517	5.5
		2: no	15,156	94.6	15,476	94.5	12,883	94.5	43,515	94.5
PHCDV2W	Had a health professional visit in the past 2 weeks	1: yes	2666	16.6	3014	18.4	2552	18.7	8232	17.9
		2: no	13,362	83.4	13,355	81.6	11,083	81.3	37,800	82.1

**Note**: Freq. represents the absolute frequency distribution for the qualitative variables; while Rel. Freq. represents the relative frequencies in (%).

## 2 Experimental design, materials, and methods

This section describes how the key health care market and labor market outcomes presented in the previous section were constructed using the information provided in the raw data files. All data treatment, variable reformulation, and descriptive statistics are implemented within the R Statistical Software [6]; ( see the attached computer codes, along with [4] for further details).

### 2.1 Access to health care

Access to health care is measured as a binary indicator (PNMED12M) In the raw NHIS data. It is the outcome of the question: “During the past 12 months, was there any time when the individual needed medical care, but did not get it because the individual couldn׳t afford it?” as such, PNMED12M takes the value 1 if yes, and 2 if No. In this data, we create the binary variable “Access” which takes the value 0 if PNMED12M =1, and the value 1 if PNMED12M=2. This variable “Access” is intended to describe the “extensive margin” of health care demand.

### 2.2 Usage of health care

Utilization of health care is also measured as a binary indicator (P10DVYR) In the raw NHIS data. It is the outcome of the question: “During the past 12 months, did the person receive care from doctors or other health care professionals 10 or more times? Does not include telephone calls”. Therefore, the variable P10DVYR takes the value 1 if yes and 2 if No. In the treated data, we create the binary variable “Utilization” which takes the value 1 if P10DVYR =1, and the value 0 if P10DVYR = 2. This variable “Utilization” is intended to capture the “Intensive margin” of health care demand.

### 2.3 Level of health care expenditure

Family level of health expenditure is measured as a nominal variable (HCSPFYR) with six modalities in the raw NHIS data. It is the outcome of the question: “The next question is about money that [you have/your family has] spent out of pocket on medical care. We do not want you to count health insurance premiums, over the counter drugs, or costs that you will be reimbursed for. In the past 12 months, about how much did [you/your family] spend for medical care and dental care?”. As such, the variable HCSPFYR =0 if amount spent is zero, HCSPFYR = 1 if less than $500, HCSPFYR = 2 if [$500; $1999], HCSPFYR =3 if [$2000; $2999], HCSPFYR =4 if [$3000; $4999], and HCSPFYR = 5 if $5000 or more. In the treated data, we create the variable “FamMedExp” which combines the modalities 3, 4 and 5 of HCSPFYR into a single category, such that “FamMedExp” is also multinomial with 4 modalities represented as: FamMedExp = 0 if HCSPFYR =0, FamMedExp = 1 if HCSPFYR =1, FamMedExp = 2 if HCSPFYR =2, FamMedExp = 3 if HCSPFYR =3, FamMedExp = 4 if HCSPFYR = 4 or more. This variable “FamMedExp” is intended to capture the “Out-of-pocket” non-reimbursed cost of health care demand.

### 2.4 Labor supply

Individual labor supply is measured by employment status and weekly hours of work. Employment status is a multinational variable with 5 modalities and captured by (DOINGLWP) in the raw NHIS; It is the outcome of the question: “Which of the following was the individual doing LAST WEEK?”. On the other hand, weekly hours of work is a non-negative continuous variable and represented by (WRKHRS2) in raw NHIS; it is the outcome of the question: “How many hours did the individual work last week at all jobs or businesses?” or “How many hours does the individual usually work weekly at all jobs or businesses?”.

The different modalities of employment status are DOINGLWP = 1 if working for pay at a job or business; DOINGLWP = 2 if with a job or business but not at work; DOINGLWP = 3 if looking for work; DOINGLWP = 4 if working, but not for pay, at a family-owned job or business;.

DOINGLWP= 5 if not working at a job or business and not looking for work. In the treated data, we use this variable to create the binary indicator “WorkStat”, as the individual׳s employment status which takes the values WorkStat = 1 if DOINGLWP = 1 (or working for pay at a job or business), and WorkStat = 0 otherwise. This variable “WorkStat” is used to describe the “extensive margin” of individual׳s labor supply. For the “intensive margin” of labor supply we use the binary variable “Full-time” to describe whether or not an individual is working part-time (WRKHRS2 less than 40 h a week) or full-time (WRKHRS2 greater than or equal to 40 hours a week).

### 2.5 Inter-generational co-residence

To obtain the main independent variable describing individual׳s inter-generational co-residence status, we use the variable (PARENTS) in the NHIS. This variable is polytomous with 4 levels, and is the outcome of the question: “Which of your Parent(s) is (are) present in the family?”; therefore, the 4 modalities of the variable are: PARENTS = 1 if mother, no father; PARENTS =2 if father, no mother; PARENTS =3 if mother and father; and PARENTS = 4 if neither mother nor father. For our analysis the Inter-generational co-residence status is captured by the binary indicator variable “Coresid” which takes the values Coresid = 0 if PARENTS =4; and Coresid= 1 if (PARENTS = 1, or 2, or 3). The interpretation of this variable is that an individual is in inter-generational co-residence if he/she lives in the same household with at least one of his parents (coresid=1), and conversely (coresid = 0) if neither the mother nor the father lives in the household. Here, mother and father can include biological, adoptive, step, and foster relationships, but not legal guardians.
