# Supplementary material for: Pooled cross-sectional sample data of the 2015, 2016, 2017 National Health Interview Surveys for studying the determinants of health care market and labor market outcomes in post Affordable Care Act USA
Source: Data Brief. 2018 Oct 30;21:1526–32. doi: 10.1016/j.dib.2018.10.130 (PMC6240666; doi:10.1016/j.dib.2018.10.130)
Supplement: Supplementary file 4 — Supplementary material [file mmc4.pdf]

The relative influence of inter-generational co-residence on healthcare market and labour market outcomes in post-Affordable Care Act USA

Ibrahim Niankara

College of Business,
Al Ain University of Science and Technology,
P.O. Box 112612, Abu Dhabi, UAE
Fax: +97124444304
Email: ibrahim.niankara@aau.ac.ae

Abstract: This paper investigates the effects of inter-generational co-residence on healthcare market, and labour market outcomes in post-Affordable-Care Act (ACA) USA. The analytical strategy involves not only looking at the gender differences in co-residence, but also accounting for co-residence endogeneity using a switching regression approach. This novel approach on the topic defines co-residence as an endogenous selection process using a binary probit equation, and modelled jointly with the extensive margins and intensive margins in the two markets, and estimated using penalised maximum likelihood methods. The results show that co-residence increases healthcare expenditure by 56.7% among females, while this figure increases to 74.2% among males. In addition, co-residing individuals, while having a 69.7% higher annual family healthcare expenditure are 1.22 times more likely to access healthcare, but 31% less likely to use healthcare intensively during the year. In the labour market, co-residence is found to reduce significantly hours of weekly labour supplied by 41% for females, and 55.6% for males. Furthermore, co-residing individuals, while not significantly different in their likelihood of labour force participation, are 1.52 times less likely to work full time once they decide to participate, and also spend about 55.4% less time working in the labour market in post-ACA USA.

Keywords: Affordable Care Act; ACA; access to care; co-residence; labour supply; medical expenditure.

Reference to this paper should be made as follows: Niankara, I. (xxxx) 'The relative influence of inter-generational co-residence on healthcare market and labour market outcomes in post-Affordable Care Act USA', *Global Business and Economics Review*, Vol. X, No. Y, pp.xxx–xxx.

Biographical notes: Ibrahim Niankara has a PhD in Economics with a field of specialisation in international economics, health economics and applied econometrics. He is a published author in several journals including the *Journal of Clinical Medicine and Therapeutics*, the *Journal of Global Business and Trade*, the *International Journal of Economics and Business Research*, the *International Journal of Behavioral and Health Care Research*. His research has been presented at numerous prestigious meetings in business and health economics, including annual meetings of the American Economic Association, the Business and Economics Society International and the International Health Economics Association of which he is an active member.

Copyright © 20XX Inderscience Enterprises Ltd.

1 Introduction

Although many factors shape global residence patterns, modernisation theory emphasises the role of economic development and industrialisation as the primary determinants of trends in inter-generational co-residence (Peng, 2009; Bordoloi and Das, 2017). These relationships typically involve both affective ties and more instrumental forms of support such as financial resources or child care (Mandell and Kim, 2017). In reporting on inter-generational co-residence in the USA between 1850 and 2000, Ruggles (2007) notes a historical decline which was justified using two major hypotheses: The affluence hypothesis and the economic development hypothesis.

The affluence hypothesis as the consensus view of social scientists in recent decades, posits that rising economic resources of the aged increasingly allowed them to maintain independent residences (McGarry and Schoeni, 2000), while the economic development hypothesis, embraced by earlier generations of theorists and policymakers, contends that rising wage-labour opportunities and the declining importance of agricultural inheritance reduced the incentives for members of the younger generation to remain in their parents' homes (Rosenfeld, 2006). A more recent evaluation of inter-generational co-residence patterns in the USA by Ruggles (2011) suggests its probable reciprocal nature between generations than principally a means of old-age support. A view also shared by Grundy (2005).

As a social phenomenon however, inter-generational family relationships such as co-residence reflect adaptations to the prevailing state of economic and cultural conditions (Compton and Pollak, 2015). And as recent studies show, inter-generational relationships between adult children and their parents are increasingly becoming important to Americans not only because of rising marital instability and broader demographic shifts (Swartz, 2009), but also because of economic crises (Keene and Batson, 2010).

In fact, the global financial crisis of 2008 which originated in the USA, put a major dip on wage-labour opportunities globally (Cho and Newhouse, 2013; Khanna and Newhouse, 2013; Bruno et al., 2017) including in the USA (Verick, 2009) and thus contributed to weakening both the economic development hypothesis and the affluence hypothesis of reduced inter-generational co-residence in recent years (Keene and Batson, 2010). A report on Income, Poverty and Health Insurance Coverage in the United States based on information collected in the 2011 and earlier Current Population Survey Annual Social and Economic Supplements (CPS ASEC) conducted by the US Census Bureau (Proctor, 2016) shows that between 2009 and 2010, real median household income declined, while both poverty rate and the number of people without insurance increased. Young people were far more affected by the employment crises than the elder; long-term unemployment for young workers were significantly harmed resulting in 'discouraged workers' effects and social exclusion from labour markets (Choudhry et al., 2012; Sorgente and Lanz, 2017). As a result, several policies were enacted in the USA to mitigate the societal effects of the global recession (Verick and Islam, 2010). Among those policies were the Affordable Care Act (ACA) (Harrington, 2010; Sommers et al., 2012), which to this date is recognised as the biggest health policy change in the US history after Medicare (Shaw et al., 2014) and Social Security which have contributed to the rise of the affluence hypothesis and the economic development hypothesis of reduced inter-generational co-residence in the USA.

In Europe, Courtin and Avendano (2016) shows that in the context of high unemployment rates during the great recession of 2008, increased inter-generational exchange between adult children and older parents in the form of co-residence had positive mental health effects on older parents. In the USA however, although Keene and Batson (2010) suggests that inter-generational co-residence responds to national economic conditions, no study to the best of our knowledge reports on the influence of inter-generational co-residence on healthcare market and labour market outcomes in the period post-ACA enforcement. Therefore, the aim of the current analysis is to describe how the changes in the US health policy framework through the ACA have been affecting the dynamics in the US healthcare market and labour market. The general question it seeks to address is: "Does living under the same roof with at least one parent matters in healthcare market and labour market behaviours in post-ACA USA?"

More specifically:

Q1 Is the likelihood of inter-generational co-residence the same for males and females in the USA between 2015 and 2016?

Q2 Does inter-generational co-residence significantly affect healthcare access, usage and expenditure in the USA between 2015 and 2016?

Q3 Are the effects of inter-generational co-residence on healthcare access, usage and expenditure the same for males and females in the USA between 2015 and 2016?

Q4 Does inter-generational co-residence significantly affect labour market participation, full-time work status and time spent working in the USA between 2015 and 2016?

Q5 Are the effects of inter-generational co-residence on labour market participation, full-time work status and time spent working the same for males and females in the USA between 2015 and 2016?

The maintained null hypotheses with respect to the above questions are:

$H_0(1)$ There are no significant differences in likelihood of inter-generational co-residence between males and females in post-ACA USA.

$H_0(2)$ Inter-generational co-residence has no bearing in healthcare access, usage and expenditure in post-ACA USA.

$H_0(3)$ There are no gender inequalities in inter-generational co-residence effects on healthcare market outcomes in post-ACA USA.

$H_0(4)$ Inter-generational co-residence has no bearing on labour market participation, full-time work status and time spent working in post-ACA USA.

$H_0(5)$ There are no gender inequalities in inter-generational co-residence effects on labour market outcomes in post-ACA USA.

In order to test the above five hypotheses and answer the raised questions, we use a pooled cross-sectional sample of the 2015 and 2016 National Health Interview Surveys (NHIS), since 2015 marks the starting point of ACA penalty enforcement since its introduction in 2010. Because different states have been implementing the act in different

ways, we anticipate regional heterogeneity in ACA effects on healthcare market and labour market outcomes in the USA.

The rest of the analysis is organised as follows: Section 2 presents the legal, socio-political and contextual framework of the study; Section 3 describes the behavioural economic model, while Section 4 presents the econometric framework and model identification strategies. Section 5 proceeds to describe the data, while Section 6 presents and discusses the results and finally, Section 7 concludes the analysis.

2 Legal, socio-political and contextual background

2.1 The ACA

The Patient Protection and ACA, more commonly referred to as 'Obamacare' is the healthcare law passed by Congress on March 3, 2010 during the administration of President Barack Obama, with the aim of ensuring that most Americans can receive or buy health insurance (Harrington, 2010). Phased in over four years from 2011 to 2015, ACA constituted what is widely considered the most sweeping change to the US healthcare system since Medicare and Medicaid were created in 1965 (Harrington, 2010; Even and Macpherson, 2016).

Together, the individual mandate and company mandate of the act changed not only healthcare sector dynamics, but also labour market dynamics in the USA. In fact, the act allows insurance coverage of preventive care such as mammograms, checkups and screenings (Koh and Sebelius, 2010), which should grant more access and usage of preventive healthcare (Bauer et al., 2014). It also made gender-based premium discrimination illegal, allowing women to pay the same health insurance premium costs as men 'ceteris paribus' (Shaw et al., 2014).

The dependent coverage provision of the act requires insurers to allow dependents to remain under parental insurance coverage until they turn 26 (Barbaresco et al., 2015), while the individual mandate requires each legal resident of the USA to have some form of health insurance or face penalties (fees) by year 2015; The fee structure is $95 per year for any uninsured adult (or 1% of annual income), for children this fee is reduced to $47.5 per child per year and to not exceed $285 per year for an entire family. The act also led to the creation of the health insurance market place (HIMP), allowing individuals to shop for affordable coverage (Pollitz et al., 2015). In regards to the company mandate, the act through its Small Business Health Option Program (SHOP) provides tax credits for business that acquire coverage for their employees and imposes the 'employers shared responsibility payment' to large companies (50 or more employees) that fail to provide 'a minimum value standard' to their employees by the year 2015.

2.2 Health market outcomes in the USA

Inter-generational co-residence effect on US healthcare market is assessed in terms of its influence on access to, usage of and expenditure on healthcare in the USA. Below, we provide a brief description of the literature on these three aspects of the US healthcare market.

2.2.1 Access and usage of healthcare in the USA

Prior to ACA, data shows that young adults represented the group with the highest rate of uninsured in the USA at 29% among individuals ages 18–24 and 27% among those 25–34, compared to 19% for 35–44 year olds and 14% for 45–64 year olds (DeNavas-Walt, 2010). Health insurance coverage however, usually improves the potential to access preventive health services (Koh and Sebelius, 2010; Cohen and Martinez, 2014; Christopher and Caruso, 2015), while access to preventive recommended health services reduce the risks of chronic diseases (Koh et al., 2011), which represent the main causes of poor health, disability and death and account for most of healthcare expenditures in the USA (Bauer et al., 2014).

Early reports of ACA effects suggest that the dependent coverage provision increased the likelihood of young adults' health insurance coverage (Proctor, 2016), access to primary care (Wallace and Sommers, 2015), excellent self-assessed health (Sommers et al., 2015) and low body mass index (Barbaresco et al., 2015). The gains for adults ages 19–25, continued to grow throughout 2011, with the largest gains recorded for unmarried adults, non-students and men (Sommers et al., 2012).

Healthcare services utilisation in a population is affected by several factors including:

1 socio-economic status,

2 physician supply

3 risk behaviours among members

4 health status

5 policies and beliefs (Morreale, 1998; Rosenstock, 2005).

Focusing on the relative influence of the last factor, with regards to the usage of healthcare in the USA, ACA enhances Medicare coverage for preventive services by eliminating patient cost-sharing and introducing an 'annual wellness visit' (DiSantostefano, 2011; Dallmann-Papke and Scott, 2016) free-of-charge for seniors (Jensen et al., 2015). Although racial and regional disparities exist among young adult trauma patients (Scott et al., 2015), overall, the young adult mandate has contributed to improve the use of inpatient medical care (Antwi et al., 2015a; Han et al., 2015), while reducing unnecessary emergency department visits (Antwi et al., 2015b) in this age group. With more than two-thirds of the US population overweight or obese (Cecchini and Sassi, 2015), prevention interventions through ACA provides substantial savings potentials in the long-term by significantly reducing the use of healthcare services and expenditure.

2.2.2 Expenditure on healthcare in the USA

Prior to the ACA, in 2004 the USA spent \$1.9 trillion or 16% of its gross domestic product (GDP) on healthcare; averaging to about \$6,280 for each man, woman and child (Stanton and Rutherford, 2006). A comparative analysis of health expenditure relying on OECD's System of Health Accounts (SHA) data by Lorenzoni et al. (2014) shows that during the period of 2000 to 2011, the USA had the highest total and public health expenditure per person and share of GDP spent on healthcare, of the examined countries. Out-of-pocket (OOP) health expenditure per person was higher than in other

high-spending countries (except Switzerland) (Baltagi and Moscone, 2010). The quality of care especially in the primary care sector, based on OECD healthcare quality indicators, did not seem to justify however the observed higher health expenditure in the country (Jaffe and Frieden, 2014). Lorenzoni et al. (2014) however, report a sharp decline in health expenditure growth per person in the USA from 2000 to 2011, after adjusting for inflation. Although by 2012, health spending reached $2.8 trillion or $8915 per person and accounted for 17.2% of GDP (Jaffe and Frieden, 2014). Prevention interventions in 2010 had the potential to decrease total healthcare expenditure by up to $2 billion (Cecchini and Sassi, 2015). After additionally controlling for household income, education and employment (Chua and Sommers, 2014) reports that ACA dependent coverage provision is associated with improved protection against medical costs (a 3.7 percentage points reduction in OOP expenditure) among adults aged 19 to 25 years compared with older adults relatively unaffected by the law. Similarly, using difference-in-difference comparison between the near elderly (ages 60 to 65) and elderly enrolled in Medicare (ages 65 to 70), Allison (2017) reports a reduction in the odds of OOP and total expenditures exceeding zero for the overall sample, but not the treatment group in 2014. As of 2016, national medical expenditure amounted to $3.3 trillion or about $10,500 per person, for the 325 million people living in the USA and represented about 18% of the country's GDP (Phelps, 2017).

2.3 Inter-generational co-residence and labour market outcomes

Inter-generational co-residence effect on labour market outcomes is assessed in terms of individuals' labour supply decisions that is, the decision of whether or not to work [the extensive margin (EM)] and the decision of how many hours to work [intensive margin (IM)]. Several studies have looked at this effect especially for the case of female labour force participation. For example, outside of the USA, using data from China, Shen et al. (2016) reports that women co-residing with their parents are 27.9 percentage points more likely to work than those living apart, while women living with their parents in the same neighbourhood are 34.9 percentage points more likely to work than those living in a different neighbourhood. They also found that on average, co-residence or nearby residence with parents significantly increases women's work time by 20–26 hours per week. Conversely, Landmann et al. (2017) finds that co-residence, although insignificant, reduces female labour supply in Kyrgyzstan. In the USA, using a pooled cross-sectional sample compiled from 11 years of the panel study of income dynamics, Kolodinsky and Shirey (2000) reports significantly different labour market outcomes for inter-generational co-residing females compared to their non-co-residing counterparts. Similarly, using the National Survey of Families and Households and the public use files of the US Census, Compton and Pollak (2014) found that the predicted probability of employment and labour force participation is 4–10 percentage points higher for married women with young children living in close proximity to their mothers or their mothers-in-law compared with those living further away.

Because of the interconnected relationships between health insurance, health and labour market outcomes (Madrian, 2006; Rutledge, 2016), the federal dependent coverage provision of the health insurance mandate has the potential to not only re-enforce inter-generational family relations, but also distort labour market activity and time allocation (Shrestha and Lenhart, 2015; Dolan, 2016; Lenhart and Shrestha, 2016; Duggan et al., 2017). In fact, relying on evidence from the US time use survey, Colman

and Dave (2018) reports that ACA dependent coverage mandate decreases young adults' labour supply, with the increased free time used for socialising and educational and job-search activities. A similar decrease in labour supply is recorded by Depew (2015) at the IM. On the other hand, using a triple-difference strategy and tax data to examine the impact of ACA on labour market outcomes, Heim et al. (2015) reports no substantial change. Similarly, in reviewing evidence over the five years of ACA implementation, Schoen (2016) concludes that the ACA has had no net negative economic impact and, in fact, has likely helped to stimulate growth by contributing to the slower rise in healthcare costs. Overall however, Gaudette et al. (2016) reports an inconclusive effect through a literature review on the lifetime consequences of early and midlife access to health insurance on labour market outcomes.

3 The behavioural economic model

Our economic model of inter-generational co-residence, healthcare market and labour market outcomes is grounded on the discrete choice modelling framework (Niankara, 2018), derived under random utility theory (Azari et al., 2012; Manski, 1977). For this, we make two different assumptions about the process generating inter-generational co-residence, allowing us to derive two distinctive models where one is a special case of the other and obtained through a one dimensional restriction. The first model is derived under the assumption of endogenous co-residence effects yielding a trivariate sample selection model (TSSM) with a recursive system of three equations, while the second model is derived under the assumption of exogenous co-residence effect producing a bivariate sample selection model (BSSM) with a recursive system of two equations.

3.1 The random utility model with endogenous selection: TSSM

In the endogenous formulation, we assume that the individual (agent) contemplates participation in two markets M_i for (i = 1, 2), where M_1 = healthcare market and M_2 = labour market. For each market M_i, the individual is faced with three choice situations (or decisions) D_j for (j = 0, 1, 2), where D_0: should I co-reside with my parents or not? (the selection process), D_1: should I participate in the market or not? (EM) and D_2: should I participate intensely or not? (IM). For each decision D_j, the individual is faced with two alternatives A_k for (k = 0, 1), where A_0 = no and A_1 = yes.

Therefore, the individual's problem consists of choosing alternative A_k in choice situation D_j in market M_i for all (i = 1, 2), (j = 0, 1, 2) and (k = 1, 2) based on the relative expected utility of the choices. That is, by the expected utility theory (Mongin, 1997), there exist an additive random utility function (Hey and Orme, 1994; Niankara, 2017):

$$U_{ijk} = V_{ijk} + \varepsilon_{ijk} \quad (1)$$

where U_{ijk} is the utility of the kth alternative in the jth choice situation, within the ith market. V_{ijk} represents the deterministic components of the utility function, while ε_{ijk} represent the random components. Abstracting from the alternatives index k, this function can be rewritten more explicitly, for all (i = 1, 2) and (j = 0, 1, 2) as:

$$U_{ij0} = V_{ij0} + \varepsilon_{ij0}$$
$$U_{ij1} = V_{ij1} + \varepsilon_{ij1} \tag{2}$$

Therefore, we observe the outcome $y_{ij} = 1$, if and only if $U_{ij1} > U_{ij0}$, that is if alternative A_1 has the highest utility in the jth choice situation, within the ith market. Since the utility function has random components, this event is also random with probability represented as:

$$\begin{aligned} Pr\left[y_{ij} = 1\right] &= Pr\left[U_{ij1} > U_{ij0}\right] \\ &= Pr\left[V_{ij1} + \varepsilon_{ij1} > V_{ij0} + \varepsilon_{ij0}\right] \\ &= Pr\left[\varepsilon_{ij0} - \varepsilon_{ij1} < -\left(V_{ij0} - V_{ij1}\right)\right] \\ &= F\left(V_{ij0} - V_{ij1}\right) \end{aligned} \tag{3}$$

where $F(.)$ is the multivariate cumulative distribution function of the error differences $(\varepsilon_{ij0} - \varepsilon_{ij1})$ and giving:

$$Pr\left[y_{ij} = 1\right] = F\left(X_j'\beta_{ij}\right) \text{ if } V_{ij0} - V_{ij1} = X_j'\beta_{ij} \tag{4}$$

Furthermore, if we assume independence of the two markets, then equation (4) can be rewritten more explicitly as

$$\begin{aligned} Pr\left[y_{1j} = 1\right] &= F\left(X_j'\beta_{1j}\right) \text{ if } V_{1j0} - V_{1j1} = X_j'\beta_{1j} \\ Pr\left[y_{2j} = 1\right] &= F\left(X_j'\beta_{2j}\right) \text{ if } V_{2j0} - V_{2j1} = X_j'\beta_{2j} \end{aligned} \tag{5}$$

where $Pr[y_{1j} = 1]$ and $Pr[y_{2j} = 1]$ represent the choice probabilities in the healthcare market and the labour market, respectively. Both choice probabilities are tri-dimensional since $j = 0, 1, 2$. As such, in each market, we obtain a trivariate choice probability model describing the three decisions or choice situations D_j for ($j = 0, 1, 2$). Different parametric distributions can be specified for the error differences $(\varepsilon_{1j0} - \varepsilon_{1j1})$ and $(\varepsilon_{2j0} - \varepsilon_{2j1})$, yielding different types of discrete choice models. If we assume $F(.) = \Lambda(.)$, that is the multivariate type 1 extreme value cumulative distribution, then we obtain the trivariate logistic regression model. If instead we assume $F(.) = \Phi(.)$, that is the multivariate normal cumulative distribution, then we obtain the trivariate probit model. For the rest of the analysis, we will assume the later such that for all $j = 0, 1, 2$, we have:

$$\begin{aligned} Pr\left[y_{1j} = 1\right] &= F\left(X_j'\beta_{1j}\right) = \Phi\left(X_j'\beta_{1j}\right) \text{ for the healthcare market} \\ Pr\left[y_{2j} = 1\right] &= F\left(X_j'\beta_{2j}\right) = \Phi\left(X_j'\beta_{2j}\right) \text{ for the labour market} \end{aligned} \tag{6}$$

3.2 The random utility model with exogenous selection: BSSM

The derivation of the exogenous case is similar to that of the endogenous, as previously mentioned. However, because selection exogeneity implies that the co-residence decision is no longer under the control of the individual decision maker, we get a one dimensional restriction on the choice situations j, such that we no longer have $j = 0, 1, 2$ as in the endogenous case, but instead $j = 1, 2$ only. With this restriction, the choice probabilities $Pr[y_{1j} = 1]$ and $Pr[y_{2j} = 1]$ are now two-dimensional, such that in each market we now

have a bivariate choice probability model describing the two choice situations D_j for $j = 1, 2$ as

$$\begin{aligned} Pr\left[y_{1j} = 1\right] &= F\left(X_j'\beta_{1j}\right) = \Phi\left(X_j'\beta_{1j}\right) \text{ for the healthcare market} \\ Pr\left[y_{2j} = 1\right] &= F\left(X_j'\beta_{2j}\right) = \Phi\left(X_j'\beta_{2j}\right) \text{ for the labour market} \end{aligned} \tag{7}$$

4 Econometric specification and model identification

In this section, we present two types of econometric specifications. One corresponding to the recursive trivariate probit specification (RTPS), obtained from the TSSM in Section 3.1 and the other corresponding to the recursive bivariate probit specification (RBPS), obtained from the BSSM in Section 3.2.

4.1 Recursive trivariate probit specification

The RTPS of co-residence, healthcare market and labour market outcomes is built for all $i = 1, 2$, with ($i = 1$ if healthcare market; $i = 2$ if labour market) as shown in equation (6) as follows.

The selection process or co-residence decision (*Coresid*):

$$Coresid = \begin{cases} 1 \text{ if } \left(U_{i01} - U_{i00}\right) > 0 \text{ or } \tilde{U}_{i0} > 0 \\ 0 \text{ if } \left(U_{i01} - U_{i00}\right) \le 0 \text{ or } \tilde{U}_{i0} \le 0 \end{cases} \tag{8}$$

The participation process or *EM*:

$$EM = \begin{cases} 1 \text{ if } \left(U_{i11} - U_{i10}\right) > 0 \text{ or } \tilde{U}_{i1} > 0 \\ 0 \text{ if } \left(U_{i11} - U_{i10}\right) \le 0 \text{ or } \tilde{U}_{i1} \le 0 \end{cases} \tag{9}$$

and the level of participation or *IM*:

$$IM = \begin{cases} 1 \text{ if } \left(U_{i21} - U_{i20}\right) > 0 \text{ or } \tilde{U}_{i2} > 0 \\ 0 \text{ if } \left(U_{i21} - U_{i20}\right) \le 0 \text{ or } \tilde{U}_{i2} \le 0 \end{cases} \tag{10}$$

The recursive trivariate system of additive random utilities is obtained as:

$$\begin{cases} \tilde{U}_{i0} = X_0\beta_{i0} + \tilde{\varepsilon}_{i0} \\ \tilde{U}_{i1} = \beta_{01}Coresid + X_1\beta_{i1} + \tilde{\varepsilon}_{i1} \\ \tilde{U}_{i2} = \beta_{02}Coresid + \beta_{12}EM + X_2\beta_{i2} + \tilde{\varepsilon}_{i2} \end{cases} \tag{11}$$

where $\tilde{U}_{i0}$ represent the differences in utility associated with inter-generational co-residence, $\tilde{U}_{i1}$ is the differences in utility associated with participation (EM); while $\tilde{U}_{i2}$ represent the differences associated with intensive participation (IM). For all $i = 1, 2$ independents and $j = 0, 1, 2$ dependents, equation (11) can be rewritten more succinctly as:

$$\tilde{U}_{ij} = X_j\beta_{ij} + \tilde{\varepsilon}_{ij} \tag{12}$$

where $\tilde{U}_{ij}$ is a vector of utility differences, X_j is the design matrix in the jth choice situation, representing the explanatory variables in the system and $\tilde{\varepsilon}_{ij}$ is the vector of random disturbances. This general multivariate probit specification in equation (12) with latent utility differences $\tilde{U}_{ij}$ follows the trivariate normal distribution for all $i = 1, 2$:

$$\begin{pmatrix} \tilde{U}_{i0} \\ \tilde{U}_{i1} \\ \tilde{U}_{i2} \end{pmatrix} \sim N_3 \left[\begin{pmatrix} \tilde{\mu}_{i0} \\ \tilde{\mu}_{i1} \\ \tilde{\mu}_{i2} \end{pmatrix}, \begin{pmatrix} \tilde{\sigma}_{i0} & \tilde{\theta}_{i01} & \tilde{\theta}_{i02} \\ & \tilde{\sigma}_{i1} & \tilde{\theta}_{i12} \\ & & \tilde{\sigma}_{i2} \end{pmatrix} \right] \tag{13}$$

where $\tilde{\mu}_{ij}$ and $\tilde{\sigma}_{ij}$ are respectively the mean and variance for $\tilde{U}_{ij}$ while the $\tilde{\theta}_{ij}$ are scalar correlation parameters. In this formulation, each triplet of decision outcomes $(y_{ij0}, y_{ij1}, y_{ij2})$ has $2 \times 2 \times 2 = 8$ potential outcomes, $(y_{ij0} = 1, y_{ij1} = 1, y_{ij2} = 1)$, $(y_{ij0} = 1, y_{ij1} = 1, y_{ij2} = 0)$, $(y_{ij0} = 1, y_{ij1} = 0, y_{ij2} = 1)$, $(Y_{ij0} = 0, Y_{ij1} = 1, Y_{ij2} = 1)$, $(Y_{ij0} = 0, Y_{ij1} = 1, Y_{ij2} = 0)$, $(Y_{ij0} = 0, Y_{ij1} = 0, Y_{ij2} = 1)$, $(Y_{ij0} = 1, Y_{ij1} = 0, Y_{ij2} = 0)$ and $(Y_{ij0} = 0, Y_{ij1} = 0, Y_{ij2} = 0)$. The joint probability for these eight outcomes is modelled with six systematic components: the marginal probabilities $Pr[y_{ij0} = 1]$ and $Pr[y_{ij1} = 1]$ and $Pr[y_{ij2} = 1]$ and the correlation parameters $\tilde{\theta}_{i01}, \tilde{\theta}_{i02}$ and $\tilde{\theta}_{i12}$ for the three marginal distributions.

For identification purposes, we restrict the diagonal elements (variances) $\tilde{\sigma}_{ij}$, for $j = 0, 1, 2$ and $i = 1, 2$ in equation (13) to 1. Since the correlation parameters do not correspond to one of the decision outcomes, the model estimates $\tilde{\theta}_{01}, \tilde{\theta}_{02}$ and $\tilde{\theta}_{12}$ as constants by default, in each market. Hence, only the three means equations ($\tilde{\mu}_{i0}$ or the average difference in utility associated with co-residence (selection); $\tilde{\mu}_{i1}$ or the average difference in utility associated with participation (EM); $\tilde{\mu}_{i2}$ the average difference in utility associated with the intensity of participation (IM) are required. Each of these systematic components are modelled as functions of sets of explanatory variables. Identification of the above described parameters for the RTPS is achieved using penalised maximum likelihood methods as described in Wojtys et al. (2016).

4.2 *Recursive bivariate probit specification*

Since the RBPS is obtained from the BSSM as shown in Section 3.2, through a one dimensional restriction on the TSSM in Section 3.1, its specification is easily derived for all $i = 1, 2$ independents, from equation (11) as:

$$\begin{cases} \tilde{U}_{i1} = \beta_{01} Coresid + X_1 \beta_{i1} + \tilde{\varepsilon}_{i1} \\ \tilde{U}_{i2} = \beta_{02} Coresid + \beta_{12} EM + X_2 \beta_{i2} + \tilde{\varepsilon}_{i2} \end{cases} \tag{14}$$

Or more compactly for all $i = 1, 2$ and $j = 1, 2$ as:

$$\tilde{U}_{ij} = X_j \beta_{ij} + \tilde{\varepsilon}_{ij} \tag{15}$$

Parameters estimation for the bivariate utility function in equation (15) by maximum likelihood is straightforward if we make the following assumption about the correlated errors for all $i = 1, 2$:

$$\begin{pmatrix} \tilde{\varepsilon}_{i1} \\ \tilde{\varepsilon}_{i2} \end{pmatrix} \sim F_{i2}\left[\begin{pmatrix} 0 \\ 0 \end{pmatrix}, \begin{pmatrix} 1 & \tilde{\sigma}_{i12} \\ & \tilde{\sigma}_{i2}^2 \end{pmatrix}\right] \tag{16}$$

where the normalisation $\tilde{\sigma}_{i1}^2 = 1$ is used for identification purposes. Heckman (1979) two step estimator can be used to identify this bivariate system, see Cameron and Trivedi (2005, pp.547–548). In our analysis, both the bivariate probit and trivariate probit are implemented in the R Statistical Software (R Core Team, 2015), using the library (Marra and Radice, 2017).

5 Data

As previously mentioned in the introduction, the data used in this analysis is a pooled cross section of the 2015–2016 NHIS (National Center for Health Statistics and others, 2015, 2016). First initiated in 1957 by the National Health Survey Act of 1956, the NHIS has been conducted since 1960 by the National Center for Health Statistics (NCHS) (Blackwell et al., 2014). Although redesigned after every decennial census, NHIS is a cross-sectional household, multistage probability sample survey conducted annually by interviewers of the US Census Bureau for the Centers for Disease Control and Prevention's National Center for Health Statistics. Further details on the current sampling design are found in the NCHS report (Parsons et al., 2014). The target population is the civilian non-institutionalised population residing in the USA at the time of the interview.

The sample size can vary from year to year based on budgetary constraints. The normal annual sample size (i.e., the number of households and/or persons from whom data are collected and publicly released) for the prior 2015 sample design and the new 2016 sample design is about 35,000 households containing about 87,500 persons. In 2014, the sample size was augmented by approximately 27%; in 2015, by approximately 19%; and in 2016, by approximately 15% (National Center for Health Statistics and others, 2016). Each annual NHIS questionnaire (also called the core) consists of four main components: household composition section, family core, sample adult core and sample child core. The data used in the current analysis is from the sample adult core, which obtains additional information on health conditions, activity limitations, health behaviours and access to and use of healthcare services from one randomly selected adult (the 'sample adult') in the family.

The publicly released data files (also called 'public use data files') for the 2015 NHIS contain data for 41,493 households containing 103,789 persons in 42,288 families, while the 2016 data concerns 40,220 households containing 97,169 persons in 40,875 families. The conditional response rate for the 2015 sample adult component is 79.7%, which is calculated by dividing the number of completed sample adult interviews (33,672) by the total number of eligible sample adults (42,270). The unconditional or final response rate of 55.2% is calculated by multiplying the conditional rate of 79.7% by the final family response rate 69.3% (National Center for Health Statistics and others, 2015). Similarly, the conditional response rate for the sample 2016 adult component is 80.9%, which is calculated by dividing the number of completed sample adult interviews (33,028) by the total number of eligible sample adults (40,848), while the unconditional or final response rate of 54.3% is calculated by multiplying the conditional rate of 80.9% by the final family response rate 67.1% (National Center for Health Statistics and others, 2016).

After variables selection, data treatment and accounting for missing information, the pooled cross-sectional panel data used in the final analysis contains a total of 32,397 observations, 16,028 of which are from the 2015 sample adult component and the remaining 16,369 from the 2016 sample adult component. Below is the description of the selected variables for our analysis.

5.1 Access to healthcare

Individual's access to healthcare is measured as a binary indicator (PNMED12M) in the NHIS data. It is the outcome of the question: "During the past 12 months, was there any time when the individual needed medical care, but did not get it because the individual couldn't afford it?" as such, PNMED12M takes the value 1 if yes and 2 if no. For our analysis, we create the binary variable 'access' which takes the value 0 if PNMED12M = 1 and the value 1 if PNMED12M = 2. This variable 'access' is intended to describe the 'EM' of healthcare demand in our analysis.

5.2 Utilisation of healthcare

Individual's utilisation of healthcare is also measured as a binary indicator (P10DVYR) in the NHIS data. It is the outcome of the question: "During the past 12 months, did the person receive care from doctors or other healthcare professionals 10 or more times? Does not include telephone calls." Therefore, the variable P10DVYR takes the value 1 if yes and 2 if no. For our analysis, we create the binary variable 'utilisation" which takes the value 0 if P10DVYR = 1 and the value 1 if P10DVYR = 2. This variable 'utilisation' is intended to capture the 'IM' of healthcare demand in our analysis.

5.3 Level of healthcare expenditure

The family's level of health expenditure is measured as a nominal variable with six modalities (HCSPFYR) in the NHIS data. It is the outcome of the question:

> "The next question is about money that [you have/your family has] spent out of pocket on medical care. We do not want you to count health insurance premiums, over the counter drugs, or costs that you will be reimbursed for. In the past 12 months, about how much did [you/your family] spend for medical care and dental care?"

As such the variable HCSPFYR = 0 if amount spent is zero, HCSPFYR = 1 if less than $500, HCSPFYR = 2 if [$500; $1,999], HCSPFYR = 3 if [$2,000; $2,999], HCSPFYR = 4 if [$3,000; $4,999] and HCSPFYR = 5 if $5,000 or more. For our analysis, we create the variable 'FamMedExp' which combines the modalities 3, 4 and 5 of HCSPFYR into a single category, such that 'FamMedExp' is also multinomial with four modalities represented as: FamMedExp = 0 if HCSPFYR = 0, FamMedExp = 1 if HCSPFYR = 1, FamMedExp = 2 if HCSPFYR = 2, FamMedExp = 3 if HCSPFYR = 3, FamMedExp = 4 if HCSPFYR = 4 or more. This variable 'FamMedExp' is intended to capture the 'OOP' non-reimbursed cost of healthcare demand in our analysis.

5.4 Labour supply

Individual's labour supply is measured by employment status and weekly hours of work. Employment status is a multinational variable with five modalities and captured by (DOINGLWP) in the NHIS; it is the outcome of the question: "Which of the following was the individual doing last week?" On the other hand, weekly hours of work is a non-negative continuous variable and represented by (WRKHRS2) in NHIS; it is the outcome of the question: "How many hours did the individual work last week at all jobs or businesses?" or "How many hours does the individual usually work weekly at all jobs or businesses?"

The different modalities of employment status are DOINGLWP = 1 if working for pay at a job or business; DOINGLWP = 2 if with a job or business but not at work; DOINGLWP = 3 if looking for work; DOINGLWP = 4 if working, but not for pay, at a family-owned job or business; and DOINGLWP = 5 if not working at a job or business and not looking for work. For our analysis, we use this variable to create the binary variable 'WorkStat', as the individual's employment status which takes the values WorkStat = 1 if DOINGLWP = 1 (or working for pay at a job or business) and WorkStat = 0 otherwise. This variable 'WorkStat' is used to describe the 'EM' of individual's labour supply. For the 'IM' of labour supply, we use the binary variable 'full-time' to describe whether or not an individual is working part-time (WRKHRS2 less than 40 hours a week) or full-time (WRKHRS2 greater than or equal to 40 hours a week).

5.5 Inter-generational co-residence

To obtain the main independent variable describing individual's inter-generational co-residence status, we use the variable (PARENTS) in the NHIS. This variable is multinational with four levels and is the outcome of the question: "Which of your parent(s) is(are) present in the family?"; therefore, the four modalities of the variable are: PARENTS = 1 if mother, no father; PARENTS = 2 if father, no mother; PARENTS = 3 if mother and father; and PARENTS = 4 if neither mother nor father. For our analysis, the inter-generational co-residence status is captured by the binary indicator variable 'coresid' which takes the values coresid = 0 if PARENTS = 4; and coresid = 1 if (PARENTS = 1, 2 or 3). The interpretation of this variable is that an individual is in inter-generational co-residence if he/she lives in the same household with at least one of his parents (coresid = 1) and conversely (coresid = 0) if neither mother nor father lives in the household. Here, mother and father can include biological, adoptive, step and foster relationships, but not legal guardians.

Although earlier authors have considered inter-generational co-residence as exogenous, for example in the case of US female labour market outcomes (Kolodinsky and Shirey, 2000), numerous authors have since shown its potential endogeneity and the bias involved with treating it as an exogenous factor (Emran et al., 2015, 2016; Emran and Shilpi, 2017).

One potential source of endogeneity is unobserved characteristics such as family values, relating both to individual's co-residence decisions and labour market outcomes. For example, individuals' who highly value family ties are inclined to co-reside with their parents and also to spend more time in domestic work but less time in market work (Díaz and Dolores, 2005). As a result, unobserved characteristics introduce a downward bias in the relationship between co-residence and individuals' labour supply. Another source of

endogeneity is reversed causality. Since market alternatives to services provided by co-residing parents such as childcare (Gorry and Thomas, 2017; Brady, 2018) and housekeeping (Romero, 2002; Coltrane, 2000) can be very expensive in the USA (Kimmel, 1998), married individuals who want to work are more likely to reside with their parents in order to benefit from these services as parents share the burden of household work (Lilly et al., 2007; Zamarro, 2011). In such a case, the estimated impact of co-residence on individual employment is biased upwards if co-residence is assumed exogenous.

To solve this endogeneity problem, authors have mainly relied on instrumental variable (IV) methods, Compton and Pollak (2014) in the USA, Sasaki (2002) and Oshio and Oishi (2006) in Japan, Maurer-Fazio et al. (2011) and Shen et al. (2016) in China and Landmann et al. (2017) in Kyrgyzstan. The current analysis departs from this trend by relying instead on endogenous switching methods (Lee and Porter, 1984) implemented through the RTPS as described in Section 4.1. We then proceed to test the significance of the correlation coefficients produced by the model. If they are significant, then there is indeed presence of endogeneity and the recursive trivariate probit model will be more appropriate. If instead the correlation coefficients are not significant, then co-residence would be exogenous, in which case the bivariate recursive model as described in Section 4.2 will suffice to represent the effects of inter-generational co-residence on healthcare market and labour market outcomes.

5.6 Control variables: covariates

Like any scientific study using evidence from observational data, our interests here centres on a postulated causal influence from the attributes and environment of individuals' to their responses, or observed healthcare market and labour market choices. As highlighted in the above discussion, it is assumed that the observed healthcare market and labour market choices reflect individuals' inter-generational co-residence status, as influenced by the socio-political and contextual framework conditioned by the ACA. As such, in choosing the variables to be included in the models, the question that needs to be addressed in conjunction with our proposed behavioural model is: what other factors affect individuals' healthcare market and labour market behaviours in the USA?

Table 1 Descriptive statistics for the variables used in the analysis

Table 1 Descriptive statistics for the variables used in the analysis (continued)

Numeric control variables		Units	2015 (N1 = 16,028)	2016 (N2 = 16,369)	Whole sample (N = N1 + N2 = 32,397)			
					Male (16,455)	Female (15,942)	Total male and female	
			Mean	Mean	Mean	Mean	Mean	SD
Work hours		(In a week)	42.62	41.20	43.88	38.85	41.40	12.22
Age		(In years)	44.11	44.72	44.45	44.39	44.42	12.94
Education		(In levels)	16.11	16.19	15.97	16.33	16.15	2.63
Annual premium cost		(In $100)	38.76	40.24	41.07	37.90	39.51	33.52
		Modalities/levels	*Rel. freq. (%)*	*Rel. freq. (%)*	*Rel. freq. (%)*	*Rel. freq. (%)*	*Rel. freq. (%)*	
Inter-generational co-residence status	0	No	91.3	91.6	91.5	91.5	91.5	
	1	Yes	8.7	8.4	8.5	8.5	8.5	
Access to care despite costs	0	No	3	3.3	2.6	3.8	3.2	
	1	Yes	97	96.7	97.4	96.2	96.8	
Usage ten times or more in 12 months	0	No	91.6	91.1	93.9	88.7	91.3	
	1	Yes	8.4	8.9	6.1	11.3	8.7	
Medical expenditure in past 12 months	1	[$0–$499]	33.9	32.4	33.6	32.7	33.1	
	2	[$500–$1,999]	35.6	34.6	34.6	35.6	35.1	
	3	$2,000 and more	30.5	33.1	31.8	31.7	31.8	
Work status	0	Not working	4.3	4.2	3.8	4.8	4.3	
	1	Yes, working for pay	95.7	95.8	96.2	95.2	95.7	
Worked hours per week (binary)	0	Part-time (< 40 hours)	21	22.2	13.8	29.8	21.6	
	1	Full-time (≥ 40 hours)	79	77.8	86.2	70.2	78.4	
Worked hours per week (multinomial)	1	Less than 40 hours	21.0	22.2	13.8	29.8	21.6	
	2	[40 hrs.–80 hrs.]	77.8	76.8	84.6	69.7	77.3	
	3	more than 80 hours	1.1	1.0	1.6	0.5	1.1	
Annual earnings	1	$0–$34,999	34.8	32.8	24.7	43.2	33.8	
	2	$35,000–$64,999	35.3	35.6	35.8	35.1	35.2	
	3	65,000 and +	29.8	31.6	39.6	21.7	30.7	
Year	1	2015	100	------	49.8	49.2	49.5	
	2	2016	-----	100	50.2	50.8	50.5	
Sex	1	Male	51.1	50.5	100	-------	50.8	
	2	Female	48.9	49.5	-------	100	49.2	

Source: Authors' construction based on the 2015–2016 National Health Interview Surveys (NHIS)

		Modalities/levels	Rel. freq. (%)	Rel. freq. (%)	Rel. freq. (%)	Rel. freq. (%)	Rel. freq. (%)
Race	1	White	80.8	84.7	83.8	81.7	82.8
	2	Black	10.9	8.3	8.2	11.0	9.6
	3	Asian	7.2	5.8	6.8	6.1	6.5
	4	All other races	1.1	1.2	1.2	1.1	1.2
Marital status	1	Currently married	65.3	66.2	69.1	62.2	65.7
	2	Previously married	11.9	11.9	8.3	15.6	11.9
	3	Never married	22.9	21.9	22.6	22.2	22.4
Region	1	South	33.7	33.1	32.8	34.0	33.4
	2	Northwest	16.5	17.5	16.7	17.3	17.0
	3	Midwest	23.0	23.8	23.5	23.4	23.4
	4	West	26.8	25.6	27.0	25.4	26.2
Place of birth	1	Born in the USA	82.9	86.4	83.8	85.5	84.7
	2	Born outside the USA	17.1	13.6	16.2	14.5	15.3
Citizenship	1	US citizen	94	95.2	93.8	95.4	94.6
	2	Not US citizen	6	4.8	6.2	4.6	5.4
Problems paying medical bills	1	Yes	12.6	12.2	11.3	13.6	12.4
	2	No	87.4	87.8	88.7	86.4	87.6
Medical bills paid off overtime	1	Yes	27.1	26.8	25.8	28.1	26.9
	2	No	72.9	73.2	74.2	71.9	73.1
Flexible spending account	1	Yes, have	23.4	24.3	23.4	24.3	23.8
	2	No, do not have	76.6	75.7	76.6	75.7	76.2
Physical health status	1	Excellent	33.7	32.8	33.7	32.8	33.2
	2	Very good	37.6	39.3	38.1	38.8	38.5
	3	Good	23.9	23.2	23.4	23.7	23.6
	4	Fair or poor	4.8	4.7	4.8	4.7	4.7
Limited anyway physical, mental, emotional	1	Yes	3.6	4.3	3.6	4.4	4.0
	2	No	96.4	95.7	96.4	95.6	96.0
Single service plan	1	Yes	54.5	58.5	55.1	58.1	56.5
	2	No	45.5	41.5	44.9	41.9	43.5
Hospital visit in 12 months	1	Yes	5.4	5.5	3.8	7.1	5.4
	2	No	94.6	94.5	96.2	92.9	94.6
Health professional visit in two weeks	1	Yes	16.6	18.4	14.1	21.1	17.5
	2	No	83.4	81.6	85.9	78.9	82.5

Source: Authors' construction based on the 2015–2016 National Health Interview Surveys (NHIS)

Keeping in mind that the primary goal of this empirical analysis is not to describe the determinants of individuals' healthcare market and labour market choices in the USA, but to evidence the role that inter-generational co-residence play in affecting individuals'

healthcare market and labour market choices in post-ACA USA, then our main independent variable of interest is individuals' inter-generational co-residence status. In order however to achieve the study goal, we need to also control for the effects of other covariates impacting these relationships. Table 1 provides definitions and summary statistics for all the variables used in the analysis.

6 Results and discussions

Focusing on the unconditional distribution of the variables as described in Table 1, we note that the mean time spent working per week in the pooled sample is 41.4 hours, with the men sub-sample average relatively higher at 43.88 hours than the women sub-sample average of 38.85 hours. This average working time was 42.62 hours and 41.20 hours in 2015 and 2016, respectively. Similarly, the mean age in the pooled sample is 44.42 years and is fairly stable across sex and year. Finally, looking at insurance premium, the pooled sample average premium cost is found to be $3,951, distributed across sex at $4,107 for men and $3,790 for women and across time at $3,876 in 2015 and $4,024 in 2016.

With regards to our variables of direct interest, in the pooled sample 8.5% of the respondents reported co-residing with at least one parent, against 91.5% who reported not. This distribution is fairly similar for the women and men sub-samples alike and independently of time. Turning our attention to the EMs, the pooled sample results show in the healthcare market that 96.8% of the respondents had no problem accessing care, while in the labour market 95.7% reported working for pay during the two weeks prior to the survey. Moving to the IMs, the pooled sample shows in the healthcare market that only 8.7% had used intensively (ten times or more) healthcare, while in the labour market 78.4% reported working full-time (more than 40 hours a week). With regards to resource expenditure in the process of participation in the healthcare market, 33.1% of the respondents reported having medical expenses below $500, 35.1% reported spending between $500 to $2,000, while the remaining 31.8% reported expenditure above $2,000. In the labour market however, where resource expenditure is measured in terms of hours spent working, the pooled sample results in the last column of Table 1 suggest that 21.6% of respondents worked part-time (40 hours or less per week), 77.3% worked between 40–80 hours, while only 1.1% worked over 80 hours. As a major determinant of inter-generational co-residence, annual earnings figures in Table 1 suggests that 33.8% of the respondent have annual earnings below $35,000, 35.2% have earnings between $35,000 and $65,000, while the remaining 30.7% have earnings above $65,000.The remaining summary statistics for the other covariates are provided in Table 1, which presents the means and standard deviations for the continuous variables, while relative frequency distributions are shown for the nominal variables.

In addition to the above presented unconditional distribution results, we also describe the association between variables using cross-tabulations and chi-square tests. We test not only the link between our primary variable of interest (inter-generational co-residence) and a set of nominal explanatory variables, but also its link with healthcare market outcome indicators (access, usage and levels of OOP spending in dollar) and labour market outcome indicators (work status full-time status and levels of hours spent working). The results for these tests are presented in Table 2. Except for work status, all

market outcome indicators show a p-value less than the 5% significance level, suggesting their dependence with inter-generational co-residence at a 95% confidence level.

Table 2 also presents conditional frequency distributions of co-residence with the outcomes in the healthcare market and labour market, for the men and women sub-samples and the pooled sample, respectively. Focusing on the women sub-sample results, we note that among the women co-residing with at least one parent, the greater share (97.5%) reports not having any problem accessing care, while only 2.5% reports having difficulties. With regards to healthcare usage during the same year, among co-residing women, 5.9% reports using healthcare services ten times or more during the year, against 94.1% who reports less usage. For medical health expenditure levels, among co-residing women, 27.7% show spending level below \$500, 35.6% spend between \$500 and \$2,000, while the remaining 30.2% have expenditure above \$2,000.

With respect to labour market outcomes, among co-residing women, at the EM 95.6% reports working for pay, while only 4.1% reports not working. At the IM, in the binary scale 45.5% reports working part-time (less than 40 hours a week), against 54.5% who reports working full-time (more than 40 hours). On the multinational scale of intensive labour supply, 54.1% of co-residing women reports working between 40–80 hours a week, while only 0.4% works over 80 hours a week. The conditional distributions of co-residence with the healthcare market and labour market outcomes, for the men sub-sample and the pooled sample are fairly similar to that just described for the women sub-sample as shown in Table 2. For the sake of keeping the discussion concise, the reader is directed towards Table 2 for the remaining details on the conditional distribution of co-residence with the other covariates.

Now, focusing our attention on our variables of interest and abstracting from the estimated control variables (covariates) effects in the estimated models, Table 3 gives the summary representation of the effects of inter-generational co-residence on healthcare market and labour market outcomes during 2015–2016. To arrive at these results, our analytical strategy consisted of three model estimations in each market as presented below.

In the healthcare market, we look at the effects of co-residence on access to healthcare (the EM), usage of healthcare (the IM) and expenditure on healthcare, using the women sub-sample, the men sub-sample and the pooled sample. The analytical strategy in the labour market is similar to that adopted in the healthcare market, allowing us to estimate the effects of co-residence on labour market outcomes, based on the men and women sub-samples and also the pooled sample.

Although we control for the effects of a great number of covariates in each market, the presented and discussed results will focus mainly on our primary effects of interests. Furthermore, since the effect of co-residence on labour market outcomes (labour supply) has received much more attention in the literature, especially the case of female labour supply, than its effects on healthcare market outcomes, we will keep the labour market discussions brief, while providing a much detailed account of the healthcare market results, which to the best of our knowledge has been fairly untouched in the period post-ACA enforcement.

Table 2 Chi-square test and conditional frequency distribution for inter-generational co-residence status

Table 2 Chi-square test and conditional frequency distribution for inter-generational co-residence status (continued)

	Modalities/levels		*Inter-generational co-residence*						
			Females sample		*Males sample*		*Pooled sample*		
			No	*Yes*	*No*	*Yes*	*No*	*Yes*	*Chi^2 test stat.*
Access to care despite costs	0	No	96.1	97.5	97.3	99.1	96.7	98.3	21.395***
	1	Yes	3.9	2.5	2.7	0.9	3.3	1.7	
Usage ten times or more 12 months	0	No	88.2	94.1	93.6	96.8	91.0	95.5	64.169***
	1	Yes	11.8	5.9	6.4	3.2	9.0	4.5	
Medical expenditure in past 12 months	1	[$0–$499]	33.1	27.7	34.1	27.6	33.6	27.7	52.232***
	2	[$500–$1,999]	35.6	35.6	34.6	35.3	35.1	35.4	
	3	$2,000 and more	31.3	36.7	31.3	37.1	31.3	36.9	
Work status	0	Not working	4.8	4.4	3.8	3.1	4.3	3.8	1.9105
	1	Yes, working for pay	95.2	95.6	96.2	96.9	95.7	96.2	
Worked hours per week (binary)	0	Part-time (< 40 hours)	28.3	45.5	11.8	34.5	19.9	39.9	594.32***
	1	Full-time (≥ 40 hours)	71.7	54.5	88.2	65.5	80.1	60.1	
Worked hours per weeks (multinomial)	1	Less than 40 hours	28.3	45.5	11.8	34.5	19.9	39.9	596.64***
	2	[40hrs.–80 hrs.]	71.2	54.1	86.5	64.6	79.0	59.4	
	3	More than 80 hours	0.5	0.4	1.7	0.9	1.1	0.6	
Annual earnings	1	$0–$34,999	40.6	71.9	20.7	67.1	30.5	69.5	1,750.4***
	2	$35,000–$64,999	36.5	19.8	37.0	22.1	36.8	21.0	
	3	65,000 and +	22.9	8.3	42.2	10.8	32.7	9.6	
Year	1	2015	49.1	50.0	49.7	50.8	49.4	50.4	1.032
	2	2016	50.9	50.0	50.3	49.2	50.6	49.6	

Notes: *Indicates significance at alpha of 0.05; **Significance at 0.01; and ***Significance at 0.001 for the chi-square test between inter-generational co-residence status and the nominal explanatory variables in the selection process.

Source: Authors' construction based on the 2015–2016 National Health Interview Surveys (NHIS)

Table 3 Explanatory factors of co-residency in the USA, conditional on healthcare market and labour market outcomes

			Inter-generational co-residence						
		Modalities/levels	*Females sample*		*Males sample*		*Pooled sample*		
			No	*Yes*	*No*	*Yes*	*No*	*Yes*	*Chi^2 test stat.*
Sex	1	Male	-----	-----	100	100	50.8	50.9	0.016
	2	Female	100	100	----	----	49.2	49.1	
Race	1	White	82.4	74.4	84.5	76.7	83.5	75.6	122.38***
	2	Black	10.7	14.9	8.0	9.8	8.3	12.3	
	3	Asian	5.8	9.2	6.4	11.5	6.1	10.4	
	4	All other races	1.1	1.5	1.1	2.0	1.1	1.7	
Marital status	1	Currently married	66.0	21.2	73.8	19.7	70.0	20.4	4,256.3***
	2	Previously married	16.1	10.4	8.5	5.5	12.3	7.9	
	3	Never married	17.9	68.4	17.7	74.9	17.8	71.7	
Region	1	South	34.0	34.4	32.7	33.8	33.3	34.1	40.957***
	2	Northwest	17.1	19.0	16.8	16.1	17.0	17.5	
	3	Midwest	23.9	17.6	23.8	20.0	23.8	18.8	
	4	West	25.0	29.0	26.7	30.1	25.9	29.6	
Place of birth	1	Born in the USA	85.9	81.3	84.0	82.5	84.9	81.9	17.593***
	2	Born outside the USA	14.1	18.7	16.0	17.5	15.1	18.1	
Citizenship	1	US citizen	95.5	94.3	93.6	95.1	94.6	94.7	0.075
	2	Non-US citizen	4.5	5.7	6.4	4.9	5.4	5.3	

Notes: *Indicates significance at alpha of 0.05; **Significance at 0.01; and ***Significance at 0.001 for the chi-square test between inter-generational co-residence status and the nominal explanatory variables in the selection process.

Source: Authors' construction based on the 2015–2016 National Health Interview Surveys (NHIS)

Table 3 Explanatory factors of co-residency in the USA, conditional on healthcare market and labour market outcomes (continued)

Co-residence	*Given healthcare market outcomes*			*Given labour market outcomes*		
Variables	*Female sub-sample (n = 15,942)*	*Male sub-sample (n = 16,455)*	*Pooled sample (n = 32,397)*	*Female sub-sample (n = 15,942)*	*Male sub-sample (n = 16,455)*	*Pooled sample (n = 32,397)*
Constant	0.561***	0.743***	0.652***	0.737***	0.883***	0.806***
	(0.141)	(0.137)	(0.097)	(0.138)	(0.883)	(0.096)
Earnings						
2 $35,000–$64,999	–0.406***	–0.614***	–0.504***	–0.356***	–0.582***	–0.467***
	(0.041)	(0.042)	(0.029)	(0.040)	(0.041)	(0.029)
3 65,000 and +	–0.426***	–0.733***	–0.593***	–0.352***	–0.675***	–0.532***
	(0.056)	(0.051)	(0.037)	(0.055)	(0.051)	(0.037)
Year 2016	0.011	0.014	0.012	0.009	0.020	0.023
	(0.034)	(0.035)	(0.024)	(0.033)	(0.035)	(0.023)
Female sex	----------	------	–0.076**	-------	------	–0.085***
	----------	---------	(0.025)	----------	--------	(0.025)
Race						
2 Black	0.026	0.002	0.017	0.042	0.014	0.019
	(0.051)	(0.061)	(0.039)	(0.050)	(0.060)	(0.038)
3 Asian	0.333***	0.533***	0.435***	0.337***	0.541***	0.448***
	(0.069)	(0.067)	(0.048)	(0.068)	(0.066)	(0.047)
4 All other races	–0.102	0.153	0.027	–0.122	0.189	0.044
	(0.154)	(0.137)	(0.102)	(0.150)	(0.135)	(0.100)
Marital status						
2 Previously married	0.619***	0.576***	0.610***	0.563***	0.578***	0.584***
	(0.053)	(0.068)	(0.041)	(0.052)	(0.067)	(0.041)
3 Never married	0.893***	0.839***	0.877***	0.864***	0.826***	0.856***
	(0.040)	(0.042)	(0.029)	(0.039)	(0.041)	(0.029)

Notes: *Indicates significance at alpha of 0.05; **Significance at 0.01; and ***Significance at 0.001 for the chi-square test between inter-generational co-residence status and the nominal explanatory variables in the selection process.
The numbers in parenthesis (.) represent the standard errors of the estimated coefficients.

Source: Author's estimation of the selection equation in the RTPS model based on the 2015–2016 National Health Interview Surveys (NHIS)

Co-residence	*Given healthcare market outcomes*			*Given labour market outcomes*		
Variables	*Female sub-sample (n = 15,942)*	*Male sub-sample (n = 16,455)*	*Pooled sample (n = 32,397)*	*Female sub-sample (n = 15,942)*	*Male sub-sample (n = 16,455)*	*Pooled sample (n = 32,397)*
Age	–0.040***	–0.042***	–0.040***	–0.041***	0.043***	–0.042***
	(0.002)	(0.002)	(0.001)	(0.002)	(0.002)	(0.001)
Region						
2 Northwest	0.219***	0.082	0.155***	0.197***	0.062	0.137***
	(0.050)	(0.054)	(0.037)	(0.049)	(0.053)	(0.036)
3 Midwest	–0.164***	–0.136**	–0.152***	–0.153**	–0.131**	–0.145***
	(0.050)	(0.050)	(0.035)	(0.048)	(0.049)	(0.034)
4 West	0.087*	0.053	0.069*	0.073*	0.037	0.056
	(0.045)	(0.046)	(0.032)	(0.044)	(0.045)	(0.031)
Born outside the USA	0.353***	0.423***	0.385***	0.289***	0.394***	0.341***
	(0.055)	(0.057)	(0.040)	(0.053)	(0.056)	(0.039)
Not US citizen	–0.420***	–0.794***	–0.608***	–0.371***	–0.765***	–0.571***
	(0.084)	(0.085)	(0.060)	(0.082)	(0.083)	(0.058)
Education level	–0.044***	–0.036***	–0.039***	–0.052***	–0.041***	–0.045***
	(0.007)	(0.007)	(0.005)	(0.007)	(0.007)	(0.005)
AIC	20,927.8	16,319.88	37,299.87	29,507.91	22,561.14	52,164.38
BIC	21,388.4	16,782.39	37,828.17	29,968.51	23,023.65	52,675.92

Notes: *Indicates significance at alpha of 0.05; **Significance at 0.01; and ***Significance at 0.001 for the chi-square test between inter-generational co-residence status and the nominal explanatory variables in the selection process.
The numbers in parenthesis (.) represent the standard errors of the estimated coefficients.

Source: Author's estimation of the selection equation in the RTPS model based on the 2015–2016 National Health Interview Surveys (NHIS)

6.1 The selection process

The selection process describes the determining factors in inter-generational co-residence decisions in post-ACA USA. The estimated results of this process are shown in Table 3 for the men and women sub-samples and also the overall pooled cross-sectional sample. Here, co-residence is estimated jointly with the EM and the IM in both the healthcare market and the labour market.

In Table 3 under each market, the first column presents the results of the female sub-sample, the second column the results of the male sub-sample and the third column the results of the pooled cross-sectional sample. We note that the effects of the factors affecting co-residence in the USA are fairly robust across gender and market, based on the signs and significance. Factors such as earnings, age, US citizenship and education have negative effects on men and women co-residence decisions. In fact, compared to females earning less than \$35,000, those earning between \$35,000 and \$65,000 are 40.6% (in the healthcare market) and 35.6% (in the labour market) less likely to co-reside with a least one parent, while those earning above \$65,000 are 42.6% (in the healthcare market) and 35.2% (in the labour market) less likely to co-reside with at least one parent. The age coefficient suggests also that every one year increase in the age of a female respondent reduces her likelihood of co-residing with parents by 4% (in the healthcare market) and 4.1% (in the labour market). Similarly, every one level increase in education reduces females' likelihood of co-residing with parents by 4.4% (in the healthcare market) and 5.2% (in the labour market). Furthermore, based on US citizenship status, compared to their US citizen counterparts, female non-citizens are found to be 42% (in the healthcare market) and 37.1% (in the labour market) less likely to co-reside with parents.

With respect to race, the results suggest that compared to white females, only Asian female respondents show a significant and positive difference in likelihood of co-residence with parents. In fact, they have 33.3% (in the healthcare market) and 33.7% (in the labour market) more chances of co-residing with parents. Looking at the effects of marital status, we see that compared to currently married female respondents, previously married females are 61.9% (in the healthcare market) and 57.8% (in the labour market) more likely to co-reside with at least one parent. Similarly, for never married females compared to those currently married who are 89.3% (in the healthcare market) and 82.6% (in the labour market) more likely to co-reside with parents.

Similar findings are also observed with the regional dummies, where compared to females living in the south, those in the Northwest and West are more likely to co-reside with at least one parent, while those in the Midwest are relatively less likely. More specifically, the relative likelihood of co-residence for females in the northwest compared to those in the South is 21.9% (in the healthcare market) and 19.7% (in the labour market). In the West, this relative likelihood of co-residence over the Southern females is 8.7% (in the healthcare market) and 7.3% (in the labour market). In the Midwest however the relative unlikelihood of co-residence over the southern females is 16.4% (in the healthcare market) and 15.3% (in the labour market). The place of birth seems to also matter significantly in co-residence decisions, with females born outside of the USA found to be 35.3% (in the healthcare market) and 28.9% (in the labour market) more likely to co-reside compared to those born in the USA.

The males' results suggest relatively similar effects in terms of signs and significance as those above described for the females' sub-sample. The distinction with the males

comes from the regional differences in co-residence, with no significant relative difference in the Northwest and the West compared to Southern males. Finally, focusing on the pooled cross-sectional sample results as shown in column 3 under each market, we note that the estimated coefficient values for all the factors explaining inter-generational co-residence in the USA between 2015 and 2016 are fairly stable and robust across markets. We also note that all variables retain their signs and significances as in the females and males' sub-samples; the only added difference is the effect of sex, which suggests that compared to men, women are 7.6% (in the healthcare market) and 8.5% (in the labour market) less likely to co-reside with parents. This latter result seems to indicate that the process leading to female co-residence in the USA, significantly differs from that leading to male co-residence.

6.2 Co-residence effects on healthcare market outcomes

The effects of inter-generational co-residence on healthcare market outcomes in the USA are presented on the upper panel of Table 4 for the males' sub-sample, the females sub-sample and the pooled sample. Focusing on its effect on the EM of the healthcare market, we see that co-residing with at least one parent consistently improves the likelihood of accessing healthcare in the USA. In fact, among female respondents, those co-residing with at least one parent are 1.17 times more likely to access healthcare. This figure among male respondents shows an improvement in access to healthcare by a factor of 1.21 for those co-residing, compared to their non-co-residing counterparts. Abstracting from gender differences, co-residence still impacts positively access to care by a factor of 1.22.

Looking at its effect on the IM of healthcare, we note that only in the females' sub-sample and the pooled sample is co-residence affecting significantly intensive usage (ten times or more annually) of healthcare. In fact, among female respondents, co-residence with at least one parent reduces the intensive use of healthcare by 57.1%. Similarly, its overall effect in the pooled sample suggests that inter-generational co-residence reduces by 31% individuals' intensive usage of healthcare in the USA. In terms of resource expenditure in the process of healthcare access and usage, we note that irrespective of gender inter-generational co-residence increases significantly healthcare expenditure in the USA. In fact, among females, co-residence increases healthcare expenditure by 56.7%, while this figure increases to 74.2% among males. Overall however and abstracting from gender differences, co-residence increases individuals' expenditure by 69.7%.

Moving to the estimated values of the correlation coefficients (θ_{01}, θ_{02}, θ_{12}) between the healthcare market outcome indicators as represented in equation (13), the results in the upper panel of Table 4 suggest that θ_{01} the correlation coefficient between the selection process (co-residence) and the EM (access) although not significant for the males sub-sample is negative and significant for both the females sub-sample and the pooled sample. In fact, the females sub-sample correlation coefficient is –39.6% with 95% confidence interval [–62.1%; –18.4%], while the pooled sample correlation coefficient is –4.6% with corresponding 95% confidence interval of [–55.9%; –10.9%]. These results further suggest that the unobserved factors affecting individual respondents' decisions to co-reside with at least one parent correlate negatively with those affecting their likelihood of accessing healthcare in the USA and even more so for females. Conversely, the correlation coefficient θ_{12} between the EM (access) and the IM (usage) is

consistently positive and significant across all sub-samples as shown by the coefficient values 45% (among females), 23.6% (among males) and 37.8% (in the pooled sample) and their 95% confidence intervals, which are void of zero. These later results suggest that the unobserved factors affecting individuals' access to healthcare despite costs in the USA, correlate positively with those affecting their intensive usage of healthcare (ten times or more) during a year. Conversely, since θ_{02} is consistently not significant as shown by the 95% confidence intervals which contain zero, we can safely conclude that the unobserved factors affecting respondents' decision to co-reside with parents are unrelated to those affecting their intensive use of healthcare in the USA between 2015 and 2016.

6.3 Co-residence effects on labour market outcomes

The effects of inter-generational co-residence on labour market outcomes in the USA are presented on the lower panel of Table 4 for the males' sub-sample, the females sub-sample and the pooled sample. Focusing on its effect on the EM of the labour market as shown in the first column of Table 4 under 'work status', we note that co-residence with at least one parent significantly affects work status only in the females' sub-sample. More specifically it reduces the likelihood of females' participation in the US labour market by 45% during 2015 and 2016.

Looking at its effect on the IM of the labour market as shown in the second column of the lower panel, we find that co-residence unilaterally reduces the likelihood of working full-time (more than 40 hours per week), in all the considered sub-samples. This effect varies across sex in the range of [–1.575; –1.552]. In terms of resource expenditure in the process of participating in the labour market, the results shown in the third column of the lower panel of Table 4 suggest also that inter-generational co-residence reduces significantly the number of hours supplied in the US labour market. This effect is significant at the 0.1% level and varies in the range of [41%; 55.6%] across sex and suggests that on average in 2015–2016, compared to individuals not co-residing with parents, those co-residing with at least one parent work about 55.4% fewer hours in the US labour market.

Moving to the estimated values for the correlation coefficients (θ_{01}, θ_{02}, θ_{12}) between labour market outcomes as represented in equation (13), the results are presented in columns 4, 5 and 6 of the lower panel of Table 4. These results show that θ_{01} the correlation coefficient between the selection process (co-residence) and the EM (work status) is not significant as shown by the 95% confidence intervals that do contain zero. These results further suggest that the unobserved factors affecting individual respondents' decisions to co-reside with parents are unrelated to those affecting their likelihood of participating in the US labour market between 2015–2016. Conversely however, θ_{02} is consistently positive and statistically significant as shown by the 95% confidence intervals which are void of zero, therefore we can say that the unobserved factors affecting respondents' decision to co-reside with parents correlate positively at 61.1% with those affecting their intensive participation (more than 40 hours) in the US labour market between 2015 and 2016.

Table 4 Inter-generational co-residence and US healthcare market and labour market outcomes across sex in 2015–2016

	Healthcare market outcomes			*Correlation coefficients*			*Sample size*
	Extensive margin	*Intensive margin*	*Resource expenditure*				
	Access	*Usage*	*($ spent)*	*Theta 01*	*Theta 02*	*Theta 12*	*(n)*
Inter-generational co-residence (selection process)	1.167***	–0.571**	0.567***	–0.396*	0.148	0.45*	Female sub-sample
	(0.211)	(0.168)	(0.037)	(–0.621; –0.184)	(–0.024; 0.298)	(0.313; 0.547)	(15,942)
	1.207***	0.005	0.742***	–0.185	–0.055	0.236*	Male sub-sample
	(0.307)	(0.180)	(0.038)	(–0.467; 0.183)	(–0.254; 0.162)	(0.030; 0.458)	(16,455)
	1.221***	–0.310*	0.697***	–0.346*	0.048	0.378*	Pooled sample
	(0.185)	(0.123)	(0.035)	(–0.559; – 0.109)	(–0.082; 0.164)	(0.279; 0.481)	(32,397)
	Labour market outcomes			*Correlation coefficients*			*Sample size*
	Work status	*Full-time*	*(Hours Spent)*	*Theta 01*	*Theta 02*	*Theta 12*	*(n)*
Inter-generational co-residence (selection process)	–0.450*	–1.552***	–0.410***	–0.134	0.663*	0.077*	Female sub-sample
	(0.191)	(0.074)	(0.041)	(–0.037; 0.254)	(0.584; 0.729)	(0.033; 0.126)	(15,942)
	0.0004	–1.575***	–0.556***	–0.020	0.594*	0.009	Male sub-sample
	(0.188)	(0.079)	(0.045)	(–0.186; 0.220)	(0.518; 0.674)	(–0.058; 0.071)	(16,455)
	–0.215	–1.524***	–0.554***	0.052	0.611*	0.048*	Pooled sample
	(0.138)	(0.055)	(0.030)	(–0.056; 0.213)	(0.561; 0.670)	(0.017; 0.085)	(32,397)

Notes: Numbers in parenthesis represent respectively: the standard error (s.e.) of the coefficients in the case of the outcome variables, the 95% confidence intervals (C.I.) in the case of the correlation coefficients and the sample sizes (n) in the case of the various sub-samples.
*Indicates significance at alpha of 0.05; **Significance at 0.01; and ***Significance at 0.001.

Source: Author's estimation of the recursive trivariate probit models based on the 2015–2016 National Health Interview Surveys (NHIS)

Finally, the results for θ_{12}, the correlation coefficient between the EM (work status) and the IM (full-time) is also positive and significant for the females sub-sample and the pooled sample, as shown by their 95% confidence intervals, which are both void of zero. These later results suggest that although not significant for men, the unobserved factors affecting women labour market participation in the USA during 2015–2016 correlate positively at 7.7% with the unobserved factors affecting their intensive participation (more than 40 hours per week) in the US labour market. This correlation independently of sex, using the pooled sample is statistically significant at 4.8%.

6.4 Covariates results' in the labour and healthcare markets

In the labour market, we find that in addition to inter-generational co-residence, covariates such as annual earnings, physical health status, intensive usage of healthcare during the year, marital status, age and education all impact significantly labour market outcomes (EM, IM and resource expenditure), for both men and women and overall. These results corroborate with those of previous authors that looked at inter-generational co-residence effect on labour supply, especially the case of female labour supply which has received relatively more attention in the literature over the years: Shen et al. (2016) in China, Landmann et al. (2017) in Kyrgyzstan and in the USA, Kolodinsky and Shirey (2000) and Compton and Pollak (2014).

The case of inter-generational co-residence and healthcare market outcomes however is yet to receive the same level of attention in the literature, therefore in addition to the above discussed effects of co-residence, we will provide a much detailed account of the effects of the covariates on our healthcare market outcomes of 'access', 'usage' and 'expenditure'.

Starting with the EM in the healthcare market, in terms of females' access to healthcare, we find that increased earnings level raises females' likelihood of accessing healthcare. More specifically, compared to females earning less than \$35,000 those earning annually between \$35,000 and \$65,000 are respectively 27.37% and 43.21% more likely to access the healthcare market. Similarly, the women reporting having no problem paying medical bills are found to be 97.47% more likely to access healthcare compared to those who report having problems. Also, the women that report not having any medical bills to pay over time, compared to those who do have, are 23.69% more likely to access healthcare. The results of females' access to healthcare suggest however no significant differences in females' access to healthcare based on race, region of residence, place of birth, or citizenship between 2015 and 2016. This later result seems to suggest that ACA by reorganising the US healthcare market has succeeded in reducing significantly healthcare access disparities among females based on race, region of residence, place of birth, citizenship in the country. However, the results do support a significant marital status effect, with currently married females having respectively 55.77% and 48.33% more chances of accessing healthcare, compared to their previously married and never married counterparts in the USA. Similarly, every one year increase in females' age leads to a 0.5% increase in likelihood of access to care in the USA between 2015 and 2016.

Moving to the IM in the healthcare market, in terms of females' annual usage of healthcare services between 2015 and 2016, the results suggest that reported physical health deterioration for females increases their likelihood of using healthcare intensively

(ten times or more) during the year. Similarly, in relation to regional differences in females' intensive use of healthcare between 2015–2016, only females in the Northwest show a significantly positive difference in intensive healthcare usage compared to those in the South. Also, in relation to marital status, compared to the currently married females, those that were previously married are found to be 11.75% more likely to use healthcare intensively. Education is also found to contribute positively to females' annual intensive healthcare usage at a rate of 5.15% for every one level increase in education between 2015 and 2016.

The coefficient values on the race control dummies, suggest that compared to white females, females from all other races (Black, Asian and others) are less likely to use healthcare ten times or more annually; however, this effect is only significant in the case of Black females and show that this later group is 17.69% less likely to use healthcare intensively during the year, than their white females' counterparts. In addition, the females that reported having no difficulty accessing the healthcare market because of costs are found to have 1.28 times less chances of using healthcare intensively during the year. This later result although seemingly counter-intuitive may be explained by the fact that those females with easy access to healthcare are more able to use the right amount of preventive care, allowing them to avoid unnecessary healthcare consumption during the year, from ill health. Similar observation is made about the women that reported not having a flexible spending account (FSA), which are found to be 23.21% less likely to use healthcare intensively during the year, compared to those women with FSA. Also, those females that reported not being limited in anyway (physical, mental or emotional) are found to be 80.40% less likely to use healthcare intensively during the year, compared to those that reported being limited. In addition, compared to the females with single service plans, those with multiple service plans are found to be 11.24% less likely to use healthcare services ten times or more during the year. Furthermore, age is also found to reduce significantly females' likelihood of intensive healthcare usage by 0.65% for each additional year of age. Based on the place of birth, compared to the females born outside of the USA, those born inside are 21.02% less likely to use healthcare intensively, although no significant difference is found on the ground of female citizenship status.

Overall, after accounting for the effects of the above mentioned covariates, the dependence among the three processes of co-residence with parents, accessing healthcare and using healthcare intensively, for women in the USA between 2015–2016, we found that indeed the unobserved factors affecting females' decisions to co-reside with parents correlate negatively at 39.6% (–62.1%, –18.4%) with those affecting their likelihood of accessing healthcare and unrelated to those affecting their likelihood of intensive healthcare usage. However, the dependence between access to and intensive usage of healthcare is significantly positive at 45.1% (31.3%, 54.7%). These correlation results suggest indeed the presence of endogeneity in the effects of inter-generational co-residence on healthcare market and labour market outcomes. And furthermore that the recursive trivariate probit model specification described in Section 4.1 is more appropriate for describing the effect of co-residence on healthcare market and labour market outcomes. In fact, by explicitly modelling the co-residence process using a binary equation and allowing correlations between the error term of this process with those produced by the EM and IM equations respectively, we are able to accommodate for the bias that would have otherwise prevailed have we assumed co-residence exogeneity and relied on the recursive bivariate probit model described in Section 4.2.

Detailed descriptions of the males' sub-sample and the pooled sample results for the effects of the covariates on healthcare market outcomes are left out of this paper due to space constraint, but will be reported in a subsequent research article. However, the female case described here complements the extensive literature on inter-generational co-residence and female labour market outcomes as previously mentioned.

7 Conclusions

Prior to the 2008 financial crises, many studies have shown that inter-generational co-residence as a social phenomenon had been on the decline in the USA. With the advent of the financial crises however and the resulting decrease in individual's financial autonomy due to reduced economic opportunities, many have resulted to direct and indirect family support to smooth out consumption needs. Part of this support involved not only direct financial assistance, but also other instrumental family resource sharing including cohabitation. This analysis by focusing on the period post-ACA enforcement has concerned itself with investigating the effects that inter-generational co-residence has on healthcare market and labour market outcomes in the USA during this crucial period.

In order to achieve this goal, the study relied on data from the 2015–2016 NHIS and an analytical strategy that involved not only looking at the gender differences in inter-generational co-residence effects in the two markets (healthcare market and labour market), but also accounting for co-residence endogeneity following the recent literature on co-residence. Unlike the recent literature that relied on IVs methods to resolve the endogeneity issue, the current study adopted a switching regression approach, defining inter-generational co-residence as an endogenous selection process using a binary probit equation and modelled jointly with the EMs and IMs in the healthcare market and labour market respectively. This approach resulted in a recursive trivariate probit model specification for each market and estimated using penalised maximum likelihood methods.

In relation to the questions raised and the maintained hypothesis in the introduction, the results showed that the likelihood of inter-generational co-residence is significantly different for males and females in the USA during 2015–2016. More specifically, it was found that compared to males, females are 7.6% (in healthcare market) and 8.5% (in labour market) less likely to live under the same roof with at least one parent, therefore providing enough evidence to safely reject the first hypothesis $H_0(1)$. This result seems to indicate the importance of gender sensitive social programs or community centres that take into account this gender differential in family shield to provide females with an access to resources and opportunities to prosper and thrive.

In relation to the second hypothesis, the results showed that compared to non-co-residing individuals, those living under the same roof with at least one parent, while having 69.7% higher annual family healthcare expenditure are 1.22 times more likely to access healthcare, but 31% less likely to use healthcare intensively (ten times or more) during the year. Hence, providing us with enough evidence to safely reject the second hypothesis $H_0(2)$ and potentially conclude that co-residence together with ACA dependent coverage provision improves access to health insurance and thus facilitate access to and usage of preventive care, thereby reducing the need for and burden associated with disease cure, which is relative more resources intensive.

In relation to the third hypothesis, the results showed that the effects of inter-generational co-residence on healthcare access, usage and expenditure differ between the males and females' sub-samples. In fact, although consistent effects (signs and significances) are observed for both gender in the cases of healthcare access and expenditure, in the case of usage, only in the females' sub-sample is the effect of co-residence significant. These results suggest that although co-residence reduces significantly healthcare usage among females, it has no significant impact on healthcare usage among males; as such we can safely reject the third hypothesis $H_0(3)$ and conclude the evidence is enough to suggest the presence of gender inequalities in co-residence effect on healthcare usage in post-ACA USA.

Now considering the fourth hypothesis, the results showed that compared to non-co-residing individuals, those living under the same roof with at least one parent are not significantly different in their likelihood of labour force participation, however they are 1.52 times less likely to work full time once they decide to participate and also spend about 55.4% less time working in the labour market. Therefore, supporting the idea that inter-generational co-residence significantly affects individuals' full-time work status and time spent working in post-ACA USA and hence providing enough evidence to also reject the fourth hypothesis $H_0(4)$. This result although contrasting with Heim et al. (2015) and Schoen (2016) that reported no substantial change in labour market outcomes at the IM from ACA inaction, is in line however with the most recent report by Colman and Dave (2018) using the evidence from the American Time Use Survey and Depew (2015) that both reported a decrease in labour supply at the IM in post-ACA USA. In fact, this latter group of studies report a reduction in young adults' labour supply, with the increased free time being used for socialising, furthering education and searching for better job opportunities.

With regards to the last hypothesis, the results showed heterogeneity in the effects of inter-generational co-residence on labour market participation, full-time work status and time spent working between the males and females' sub-samples. In fact, although fairly similar effects are observed in the case of full-time participation and time spent working, in terms of whether or not to participate in the labour market, only among females is the effect of co-residence significant. These results suggest that although co-residence reduces significantly the likelihood of labour force participation among females, it has no significant impact on labour force participation among males; as such we can safely reject the fifth hypothesis $H_0(5)$ and conclude the evidence is enough to suggest the presence of gender inequalities in co-residence effect on labour force participation in post-ACA USA.

Overall, the results indicate that ACA by reorganising the US healthcare market has succeeded in reducing significantly disparities in healthcare access among males and females based on race, region of residence, place of birth and citizenship. Although the ACA individual mandate made gender-based premium discrimination illegal, allowing women to pay the same health insurance premium costs as men 'ceteris paribus', much still remain to be done in terms of policy, since reasonable evidence is still found in support of not only significant differences in inter-generational co-residence status between males and females, but also significant heterogeneity in co-residence effects on healthcare market and labour market outcomes in post-ACA USA. Congress members and other US policy makers might therefore gain in focusing current and future policy efforts in reducing further the observed gender inequalities in co-residence effects on healthcare usage and labour force participation in the country.

References

Allison, J.R. (2017) *The Affordable Care Act and Prescription Drug Expenditures: A Comparison of the Near Elderly and Elderly*, PhD thesis, Georgetown University.

Antwi, Y.A., Moriya, A.S. and Simon, K.I. (2015a) 'Access to health insurance and the use of inpatient medical care: evidence from the affordable care act young adult mandate', *Journal of Health Economics*, Vol. 39, pp.171–187 https://doi.org/10.1016/j.jhealeco.2014.11.007.

Antwi, Y.A., Moriya, A.S., Simon, K. and Sommers, B.D. (2015b) 'Changes in emergency department use among young adults after the patient protection and affordable care act's dependent coverage provision', *Annals of Emergency Medicine*, Vol. 65, No. 6, pp.664–672.

Azari, H., Parks, D. and Xia, L. (2012) 'Random utility theory for social choice', *in Advances in Neural Information Processing Systems*', pp.126–134.

Baltagi, B.H. and Moscone, F. (2010) 'Health care expenditure and income in the OECD reconsidered: evidence from panel data', *Economic Modelling*, Vol. 27, No. 4, pp.804–811.

Barbaresco, S., Courtemanche, C.J. and Qi, Y. (2015) 'Impacts of the affordable care act dependent coverage provision on health-related outcomes of young adults', *Journal of Health Economics*, Vol. 40, pp.54–68 https://doi.org/10.1016/j.jhealeco.2014.12.004.

Bauer, U.E., Briss, P.A., Goodman, R.A. and Bowman, B.A. (2014) 'Prevention of chronic disease in the 21st century: elimination of the leading preventable causes of premature death and disability in the USA', *The Lancet*, Vol. 384, No. 9937, pp.45–52.

Blackwell, D.L., Lucas, J.W. and Clarke, T.C. (2014) 'Summary health statistics for us adults: national health interview survey, 2012', *Vital and Health Statistics*, Vol. 10, No. 260, pp.1–161, National Health Survey.

Bordoloi, S. and Das, R.J. (2017) 'Modernization theory', D. Richardson, N. Castree, M.F. Goodchild, A. Kobayashi, W. Liu and R. A. Marston (Eds.): in *International Encyclopedia of Geography: People, the Earth, Environment and Technology*, doi:10.1002/9781118786352.wbieg1174.

Brady M. (2018) 'The role of informal childcare in mothers' experiences of care and employment: a qualitative lifecourse analysis', Bernardi, L. and Mortelmans, D. (Eds.): *Lone Parenthood in the Life Course. Life Course Research and Social Policies*, Vol. 8, Springer, Cham, https://doi.org/10.1007/978-3-319-63295-7_11.

Bruno, G.S.F., Tanveer, M.C., Marelli, E. and Signorelli, M. (2017) 'The short-and long-run impacts of financial crises on youth unemployment in OECD countries', *Applied Economics*, Vol. 49, No. 34, pp.3372–3394.

Cameron, A.C. and Trivedi, P.K. (2005) *Microeconometrics: Methods and Applications*, Cambridge University Press, New York, USA.

Cecchini, M. and Sassi, F. (2015) 'Preventing obesity in the USA: impact on health service utilization and costs', *Pharmacoeconomics*, Vol. 33, No. 7, pp.765–776.

Cho, Y. and Newhouse, D. (2013) 'How did the great recession affect different types of workers? Evidence from 17 middle-income countries', *World Development*, Vol. 41, No. 1, pp.31–50.

Choudhry, M.T., Marelli, E. and Signorelli, M. (2012) 'Youth unemployment rate and impact of financial crises', *International Journal of Manpower*, Vol. 33, No. 1, pp.76–95.

Christopher, A.S. and Caruso, D. (2015) 'Promoting health as a human right in the post-ACA united states', *AMA Journal of Ethics*, Vol. 17, No. 10, p.958.

Chua, K-P. and Sommers, B.D. (2014) 'Changes in health and medical spending among young adults under health reform', *JAMA*, Vol. 311, No. 23, pp.2437–2439.

Cohen, R.A. and Martinez, M.E. (2014) *Health Insurance Coverage: Early Release of Estimates from the National Health Interview Survey, January–March 2014*, National Center for Health Statistics, Centers for Disease Control and Prevention.

Colman, G. and Dave, D. (2018) 'It's about time: effects of the affordable care act dependent coverage mandate on time use', *Contemporary Economic Policy*, Vol. 36, No. 1, pp.44–58.

Coltrane, S. (2000) 'Research on household labor: modeling and measuring the social embeddedness of routine family work', *Journal of Marriage and Family*, Vol. 62, No. 4, pp.1208–1233.

Compton, J. and Pollak, R.A. (2014) 'Family proximity, childcare, and women's labor force attachment', *Journal of Urban Economics*, Vol. 79, pp.72–90, https://doi.org/10.1016/j.jue.2013.03.007 and https://faculty.smu.edu/millimet/classes/eco6375/papers/compton%20pollak%202014.pdf.

Compton, J. and Pollak, R.A. (2015) 'Proximity and co-residence of adult children and their parents in the United States: descriptions and correlates', *Annals of Economics and Statistics/Annales d'E´conomie et de Statistique*, No. 117–118, pp.91–114, https://doi.org/10.1016/j.socscimed.2016.09.020.

Courtin, E. and Avendano, M. (2016) 'Under one roof: the effect of co-residing with adult children on depression in later life', *Social Science & Medicine*, Vol. 168, pp.140–149, https://doi.org/10.1016/j.socscimed.2016.09.020.

Dallmann-Papke, V. and Scott, K. (2016) 'The medicare wellness exam: a population health innovation for the older adult', *Nurse Leader*, Vol. 14, No. 5, pp.302–303.

DeNavas-Walt, C. (2010) *Income, Poverty, and Health Insurance Coverage in the United States (2005)*, Diane Publishing, Darby, PA, USA.

Depew, B. (2015) 'The effect of state dependent mandate laws on the labor supply decisions of young adults', *Journal of Health Economics*, Vol. 39, pp.123–134, https://doi.org/10.1016/j.jhealeco.2014.11.008.

Díaz, A. and Dolores, M. (2005) 'Family ties and labor supply', *Investigaciones Economicas*, Vol. 29, No. 2, pp.289–329.

DiSantostefano, J. (2011) 'Annual wellness visit for medicare beneficiaries', *The Journal for Nurse Practitioners*, Vol. 7, No. 5, pp.421–422.

Dolan, R.M.S. (2016) *Essays on the Economics of Health Insurance, Labor Markets, and Migration*, PhD thesis.

Duggan, M., Goda, G.S. and Jackson, E. (2017) *The Effects of the Affordable Care Act on Health Insurance Coverage and Labor Market Outcomes*, NBER Working Paper No. 23607 [online] http://www.nber.org/papers/w23607 (accessed 12 December 2017).

Emran, M.S. and Shilpi, F. (2017) *Estimating Intergenerational Mobility with Incomplete Data: Coresidency and Truncation Bias in Rank-based Relative and Absolute Mobility Measures*, MPRA Paper No. 80724 [online] https://mpra.ub.uni-muenchen.de/80724 (accessed 2 December 2017).

Emran, M.S., Greene, W.H. and Shilpi, F. (2015) *When Measure Matters: Coresident Sample Selection Bias in Estimating Intergenerational Mobility in Developing Countries*, MPRA Paper No. 65920 [online] https://mpra.ub.uni-muenchen.de/id/eprint/65920.

Emran, M.S., Greene, W.H. and Shilpi, F. (2016) *When Measure Matters: Coresidency, Truncation Bias, and Intergenerational Mobility in Developing Countries*, World Bank Policy Research Working Paper No. 7608 [online] SSRN: https://ssrn.com/abstract=2750204 (accessed 25 November 2017).

Even, W.E. and Macpherson, D.A. (2016) *The Affordable Care Act and the Growth of Involuntary Part-time Employment*, IZA Discussion Paper No. 9324 [online] https://ssrn.com/abstract=2663755 (accessed 25 November 2017).

Gaudette, É., Pauley, G.C. and Zissimopoulos, J. (2016) *Lifetime Consequences of Early and Midlife Access to Health Insurance: A Review*, University of Michigan Retirement Research Center (MRRC) Working Paper, WP 2016-341, Ann Arbor, MI [online] http://www.mrrc.isr.umich.edu/publications/papers/pdf/wp341.pdf (accessed 25 November 2017).

Gorry, D. and Thomas, D.W. (2017) 'Regulation and the cost of childcare', *Applied Economics*, Vol. 49, No. 41, pp.1–10.

Grundy, E. (2005) 'Reciprocity in relationships: socio-economic and health influences on intergenerational exchanges between third age parents and their adult children in Great Britain', *The British Journal of Sociology*, Vol. 56, No. 2, pp.233–255.

Han, X., Yabroff, K.R., Guy, G.P., Zheng, Z. and Jemal, A. (2015) 'Has recommended preventive service use increased after elimination of cost-sharing as part of the affordable care act in the United States?', *Preventive Medicine*, Vol. 78, pp.85–91, https://doi.org/10.1016/j.ypmed.2015.07.012.

Harrington, S.E. (2010) 'US health-care reform: the patient protection and affordable care act', *Journal of Risk and Insurance*, Vol. 77, No. 3, pp.703–708.

Heckman, J.J. (1979) 'Sample selection bias as specification error', *Econometrica*, Vol. 47, No. 1, pp.153–161.

Heim, B., Lurie, I. and Simon, K. (2015) 'The impact of the affordable care act young adult provision on labor market outcomes: evidence from tax data', *Tax Policy and Economy*, Vol. 29, No. 1, pp.133–157.

Hey, J.D. and Orme, C. (1994) 'Investigating generalizations of expected utility theory using experimental data', *Econometrica: Journal of the Econometric Society*, Vol. 62, No. 6, pp.1291–1326.

Jaffe, H.W. and Frieden, T.R. (2014) 'Improving health in the USA: progress and challenges', *The Lancet*, Vol. 384, No. 9937, pp.3–5.

Jensen, G.A., Salloum, R.G., Hu, J., Ferdows, N.B. and Tarraf, W. (2015) 'A slow start: use of preventive services among seniors following the affordable care act's enhancement of medicare benefits in the US', *Preventive Medicine*, Vol. 76, pp.37–42, https://doi.org/10.1016/j.ypmed.2015.03.023.

Keene, J.E. and Batson, C.D. (2010) 'Under one roof: a review of research on intergenerational co-residence and multigenerational households in the united states', *Sociology Compass*, Vol. 4, No. 8, pp.642–657.

Khanna, G., Newhouse, D. and Paci, P. (2013) *Fewer Jobs or Smaller Paychecks? Aggregate Crisis Impacts in Selected Middle-Income*, World Bank eLibrary, pp.17–36 [online] https://doi.org/10.1596/978-0-8213-8967-6_ch2 (accessed 10 November 2017).

Kimmel, J. (1998) 'Child care costs as a barrier to employment for single and married mothers', *The Review of Economics and Statistics*, Vol. 80, No. 2, pp.287–299.

Koh, H.K. and Sebelius, K.G. (2010) 'Promoting prevention through the affordable care act', *New England Journal of Medicine*, Vol. 363, No. 14, pp.1296–1299.

Koh, H.K., Piotrowski, J.J., Kumanyika, S. and Fielding, J.E. (2011) 'Healthy people: a 2020 vision for the social determinants approach', *Health Education & Behavior*, Vol. 38, No. 6, pp.551–557.

Kolodinsky, J. and Shirey, L. (2000) 'The impact of living with an elder parent on adult daughter's labor supply and hours of work', *Journal of Family and Economic Issues*, Vol. 21, No. 2, pp.149–175.

Landmann, A., Seitz, H. and Steiner, S. (2017) *Intergenerational Coresidence and Female Labour Supply, Beiträge zur Jahrestagung des Vereins für Socialpolitik 2017: Alternative Geld- und Finanzarchitekturen – Session: Labor Supply I*, No. B04-V1 [online] http://hdl.handle.net/10419/168282 (accessed 15 November 2017).

Lee, L-F. and Porter, R.H. (1984) 'Switching regression models with imperfect sample separation information – with an application on cartel stability', *Econometrica: Journal of the Econometric Society*, Vol. 52, No. 2, pp.391–418.

Lenhart, O. and Shrestha, V. (2016) 'The effect of the health insurance mandate on labor market activity and time allocation: evidence from the federal dependent coverage provision, *in Forum for Health Economics and Policy*.

Lilly, M.B., Laporte, A. and Coyte, P.C. (2007) 'Labor market work and home care's unpaid caregivers: a systematic review of labor force participation rates, predictors of labor market withdrawal, and hours of work', *The Milbank Quarterly*, Vol. 85, No. 4, pp.641–690.

Lorenzoni, L., Belloni, A. and Sassi, F. (2014) 'Health-care expenditure and health policy in the usa versus other high-spending OECD countries', *The Lancet*, Vol. 384, No. 9937, pp.83–92.

Madrian, B. (2006) *The US Health Care System and Labor Markets*, Technical report, National Bureau of Economic Research.

Mandell, N. and Kim, A.H. (2017) '3. Inter-generational relations in later life families', *The Sandwich Generation: Caring for Oneself and Others at Home and at Work*, p.62, Edward Elgar Publishing, Northampton, Massachusetts, USA.

Manski, C.F. (1977) 'The structure of random utility models', *Theory and Decision*, Vol. 8, No. 3, pp.229–254.

Marra, G. and Radice, R. (2017) 'A joint regression modeling framework for analyzing bivariate binary data in r', *Dependence Modeling*, Vol. 5, No. 1, pp.268–294.

Maurer-Fazio, M., Connelly, R., Chen, L. and Tang, L. (2011) 'Childcare, eldercare, and labor force participation of married women in urban china, 1982–2000', *Journal of Human Resources*, Vol. 46, No. 2, pp.261–294.

McGarry, K. and Schoeni, R.F. (2000) 'Social security, economic growth, and the rise in elderly widows' independence in the twentieth century', *Demography*, Vol. 37, No. 2, pp.221–236.

Mongin, P. (1997) 'Expected utility theory', Davis, J., Hands, W. and Maki, U. (Eds.): *Handbook of Economic Methodology*, pp.342–350, London, UK.

Morreale, M. (1998) *Fact Sheet: What Factors Can Influence Health Care Utilization?*, Nursing Effectiveness, Utilization and Outcomes Research Unit. McMaster University, University of Toronto.

National Center for Health Statistics and others (2015) *2015 National Health Interview Survey (NHIS) Public Use Data Release: NHIS Survey Description*, Division of Health Interview Statistics, National Center for Health Statistics, Centers for Disease Control and Prevention, NHIS [online] http://www.cdc.gov/nchs/nhis.htm (accessed 3 October 2017).

National Center for Health Statistics and others (2016) *2016 National Health Interview Survey (NHIS) Public Use Data Release: NHIS Survey Description*, Division of Health Interview Statistics, National Center for Health Statistics, Centers for Disease Control and Prevention, NHIS [online] http://www.cdc.gov/nchs/nhis.htm (accessed 3 October 2017).

Niankara, I. (2017) 'A risk based random utility model for population based discrete choice analysis in health economics with applications in modeling patients choices among medical treatment plans', *Global Business & Economics Anthology (GBEA)*, No. 1, pp.27–39, ISSN: 1553-1392.

Niankara, I. (2018) 'Evaluating health consumers' preferences stability through joint estimation of revealed and stated health insurance preferences data', *International Journal of Economics and Business Research*, Vol. 15, No. 2, pp.236–256.

Oshio, T. and Oishi, A.S. (2006) 'Coresidence with parents and a wife's decision to work in Japan', *The Japanese Journal of Social Security Policy*, Vol. 5, No. 1, pp.1–9.

Parsons, V.L., Moriarity, C.L., Jonas, K., Moore, T.F., Davis, K.E. and Tompkins, L. (2014) *Design and Estimation for the National Health Interview Survey, 2006–2015*, National Center for Health Statistics, Vital Health Stat 2(165)[online] https://stacks.cdc.gov/view/cdc/22466 (accessed 3 October 2017)

Peng, Y. (2009) 'Modernization theory: from historical misunderstanding to realistic development: a review of a new thesis on modernization', *Chinese Studies in History*, Vol. 43, No. 1, pp.37–45.

Phelps, C.E. (2017) *Health Economics*, Routledge, New York, NY, USA.

Pollitz, K., Tolbert, J. and Ma, R. (2015) *2015 Survey of Health Insurance Marketplace Assister Programs and Brokers*, Kaiser Family Foundation, Princeton, NJ.

Proctor, B.D. (2016) *Income, Poverty, and Health Insurance Coverage in the United States: 2010*, Report P60-256, September, Census Bureau.

R Core Team (2015) *R: A Language and Environment for Statistical Computing*, R Foundation for Statistical Computing, Vienna, Austria [online] https://www.R-project.org/ (accessed 10 October 2017).

Romero, M. (2002) *Maid in the USA*, Psychology Press, New York, NY.

Rosenfeld, M.J. (2006) 'Young adulthood as a factor in social change in the United States', *Population and Development Review*, Vol. 32, No. 1, pp.27–51.

Rosenstock, I.M. (2005) 'Why people use health services', *The Milbank Quarterly*, Vol. 83, No. 4, pp.94–124.

Ruggles, S. (2007) 'The decline of intergenerational co-residence in the United States, 1850 to 2000', *American Sociological Review*, Vol. 72, No. 6, pp.964–989.

Ruggles, S. (2011) 'Intergenerational co-residence and family transitions in the United States, 1850–1880', *Journal of Marriage and Family*, Vol. 73, No. 1, pp.136–148.

Rutledge, M.S. (2016) *The Interconnected Relationships of Health Insurance, Health, and Labor Market Outcomes*, Center for Retirement Research at Boston College, CRR WP 2016-2, https://ssrn.com/abstract=2812211 or http://dx.doi.org/10.2139/ssrn.2812211 (accessed 6 December 2017).

Sasaki, M. (2002) 'The causal effect of family structure on labor force participation among Japanese married women', *Journal of Human Resources*, Vol. 37, No. 2, pp.429–440.

Schoen, C. (2016) *The Affordable Care Act and the US Economy: A Five-year Perspective*, Commonwealth Fund [online] http://www.commonwealthfund.org/~/media/files/publications/fund-report/2016/feb/1860_schoen_aca_and_us_economy_v2.pdf (accessed 20 December 2017).

Scott, J.W., Salim, A., Sommers, B.D., Tsai, T.C., Scott, K.W. and Song, Z. (2015) 'Racial and regional disparities in the effect of the affordable care act's dependent coverage provision on young adult trauma patients', *Journal of the American College of Surgeons*, Vol. 221, No. 2, pp.495–501.

Shaw, F.E., Asomugha, C.N., Conway, P.H. and Rein, A.S. (2014) 'The patient protection and affordable care act: opportunities for prevention and public health', *The Lancet*, Vol. 384, No. 9937, pp.75–82.

Shen, K., Yan, P. and Zeng, Y. (2016) 'Coresidence with elderly parents and female labor supply in China', *Demographic Research*, Vol. 35, Article 23, pp.645–670 [online] https://www.demographic-research.org/volumes/vol35/23/ (accessed 23 February 2018).

Shrestha, V. and Lenhart, O. (2015) *The Effect of Health Insurance Mandate on Labor Market Activity and Time Allocation: Evidence from the Federal Dependent Coverage Provision*, Vol. 20, No. 1, pp.1–40, doi:10.1515/fhep-2016-0006 (accessed 12 May 2018).

Sommers, B.D., Buchmueller, T., Decker, S.L., Carey, C. and Kronick, R. (2012) 'The affordable care act has led to significant gains in health insurance and access to care for young adults', *Health Affairs*, Vol. 32, No. 1, pp.165–174.

Sommers, B.D., Gunja, M.Z., Finegold, K. and Musco, T. (2015) 'Changes in self-reported insurance coverage, access to care, and health under the affordable care act', *JAMA*, Vol. 314, No. 4, pp.366–374

Sorgente, A. and Lanz, M. (2017) 'Emerging adults' financial well-being: a scoping review', *Adolescent Research Review*, Vol. 2, No. 4, pp.255–292.

Stanton, M.W. and Rutherford, M.K. (2006) *The High Concentration of US Health Care Expenditures*, Agency for Healthcare Research and Quality, Washington, DC.

Swartz, T.T. (2009) 'Intergenerational family relations in adulthood: patterns, variations, and implications in the contemporary United States', *Annual Review of Sociology*, Vol. 35, No. 1, pp.191–212.

Verick, S. (2009) *Who is Hit Hardest during a Financial Crisis? The Vulnerability of Young Men and Women to Unemployment in an Economic Downturn*, IZA Discussion Paper No. 4359. [online] https://ssrn.com/abstract=1455521 (accessed 12 October 2017).

Verick, S. and Islam, I. (2010) *The Great Recession of 2008–2009: Causes, Consequences and Policy Responses*, IZA Discussion Paper No. 4934. [online] https://ssrn.com/abstract=1631069 (accessed 21 November 2017).

Wallace, J. and Sommers, B.D. (2015) 'Effect of dependent coverage expansion of the affordable care act on health and access to care for young adults', *JAMA Pediatrics*, Vol. 169, No. 5, pp.495–497.

Wojtys, M., Marra, G. and Radice, R. (2016) 'Copula regression spline sample selection models: the r package SemiParSampleSel', *Journal of Statistical Software*, Vol. 71, No. 6, pp.1–66 [online] https://www.jstatsoft.org/article/view/v071i06.

Zamarro, G. (2011) *Family Labor Participation and Child Care Decisions: The Role of Grannies*, RAND Labor and Population working paper series – WR-833.
